# Effectiveness of mRNA-1273 against SARS-CoV-2 Omicron and Delta variants

**DOI:** 10.1038/s41591-022-01753-y

**Published:** 2022-02-21

**Authors:** Hung Fu Tseng, Bradley K. Ackerson, Yi Luo, Lina S. Sy, Carla A. Talarico, Yun Tian, Katia J. Bruxvoort, Julia E. Tubert, Ana Florea, Jennifer H. Ku, Gina S. Lee, Soon Kyu Choi, Harpreet S. Takhar, Michael Aragones, Lei Qian

**Affiliations:** 1 Department of Research and Evaluation, Kaiser Permanente Southern California, Pasadena, CA USA; 2 Department of Health Systems Science, Kaiser Permanente Bernard J. Tyson School of Medicine, Pasadena, CA USA; 3 Moderna, Inc, Cambridge, MA USA; 4 Department of Epidemiology, University of Alabama at Birmingham, Birmingham, AL USA

**Keywords:** Viral infection, RNA vaccines

## Abstract

Severe acute respiratory syndrome coronavirus 2 (SARS-CoV-2) Omicron (B.1.1.529) variant is highly transmissible with potential immune escape. We conducted a test-negative case–control study to evaluate mRNA-1273 vaccine effectiveness (VE) against infection and hospitalization with Omicron or Delta. The large, diverse study population included 26,683 SARS-CoV-2 test-positive cases with variants determined by S gene target failure status (16% Delta and 84% Omicron). The two-dose VE against Omicron infection at 14–90 days was 44.0% (95% confidence interval, 35.1–51.6%) but declined quickly. The three-dose VE was 93.7% (92.2–94.9%) and 86.0% (78.1–91.1%) against Delta infection and 71.6% (69.7–73.4%) and 47.4% (40.5–53.5%) against Omicron infection at 14–60 days and >60 days, respectively. The three-dose VE was 29.4% (0.3–50.0%) against Omicron infection in immunocompromised individuals. The three-dose VE against hospitalization with Delta or Omicron was >99% across the entire study population. Our findings demonstrate high, durable three-dose VE against Delta infection but lower effectiveness against Omicron infection, particularly among immunocompromised people. However, three-dose VE of mRNA-1273 was high against hospitalization with Delta and Omicron variants.

## Abstract (web summary)

A test-negative case–control analysis using data from a diverse population in California, USA, demonstrates that vaccine efficacy of a three-dose regimen of the mRNA-1273 COVID-19 vaccine is reduced against infection with the Omicron SARS-CoV-2 variant in comparison to Delta, but that efficacy against hospitalization remained high for both variants.

## Main

The SARS-CoV-2 Omicron (B.1.1.529) variant that emerged in December 2021 contains multiple novel spike protein mutations, raising concerns about escape from naturally acquired or vaccine-elicited immunity^1^. Several in vitro studies reported reduced vaccine-induced neutralization activity against Omicron^2,3^. Specifically, sera from individuals vaccinated with two doses of mRNA Coronavirus Disease 2019 (COVID-19) vaccines, including mRNA-1273 (Moderna), showed substantial reductions in neutralization activity against Omicron compared to wild-type SARS-CoV-2 (refs. ^2,4,5^). However, an mRNA-1273 booster increased neutralization activity against Omicron, albeit lower than wild-type^2,3^. We previously reported high and durable VE of mRNA-1273 against infection and hospitalization from COVID-19 caused by other emerging SARS-CoV-2 variants, including Delta (B.1.617.2)^6^. Although limited data are available on real-world VE of mRNA-1273 against Omicron, an analysis of a US pharmacy-based testing program found that the likelihood of vaccination with three mRNA-1273 vaccine doses (versus unvaccinated) was significantly lower among Omicron symptomatic infections (odds ratio (OR) = 0.31) than SARS-CoV-2-negative controls^7^. Another US study during an Omicron-predominant period found that receipt of a third mRNA vaccine dose was 90% effective in preventing COVID-19-associated hospitalization^8^.

As the Omicron BA.1 sub-lineage has a deletion at positions 69–70, initial Omicron-positive specimens exhibit S gene target failure (SGTF). To provide timely results for these analyses, we used SGTF as a marker for Omicron in specimens collected during December 2021. The US Food and Drug Administration (FDA) and World Health Organization advised that SGTF from select COVID-19 RT–PCR assays, including the Thermo Fisher TaqPath COVID-19 Combo Kits, can be used as a screening method for Omicron;^9,10^ SGTF has served as a proxy in the United Kingdom for identifying Omicron^11,12^. In Southern California, where Delta was the dominant strain before Omicron^13^ and the proportion of SGTF among SARS-CoV-2-positive specimens increased from 1.2% to 94.1% from 6 December 2021 to 31 December 2021, SGTF can be used as a proxy for Omicron sub-lineage BA.1, whereas positive specimens negative for SGTF can be considered Delta. Using electronic health records (EHRs) from the Kaiser Permanente Southern California (KPSC) healthcare system in the United States, we conducted a test-negative case–control study to evaluate the VE of mRNA-1273 against infection and hospitalization with Omicron and Delta.

## Results

**Fig. 1:**
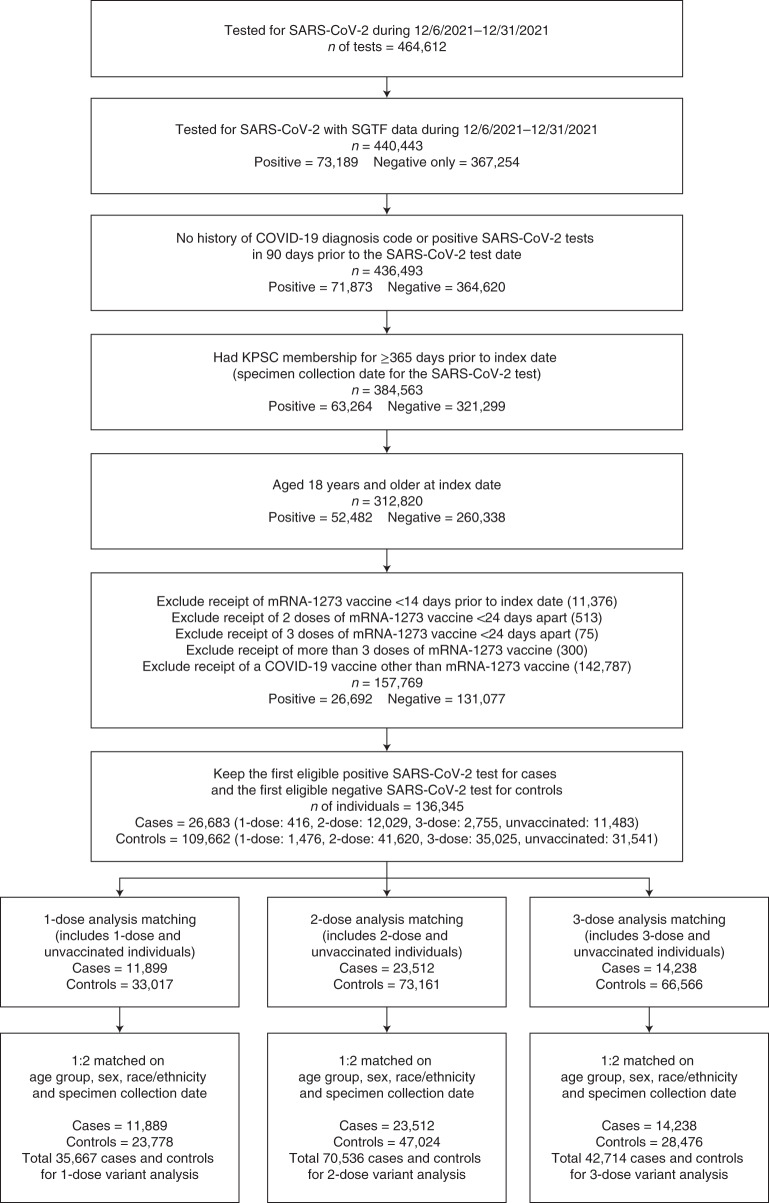
Flowchart of selection of cases and controls. Steps for selection of 26,683 cases and 109,662 controls by inclusion and exclusion criteria and subsequent matching in one-dose, two-dose and three-dose analyses.

The study included 26,683 cases with SGTF status available. Based on whole-genome sequencing (WGS) results received for a subset of 1,383 positive specimens, we confirmed that all 704 cases exhibiting SGTF were Omicron (100%), and 673 of the 679 SGTF-negative cases were Delta (99.1%), with a kappa of 0.991. The sensitivity and specificity of SGTF in predicting Omicron was 99.2% and 100%, respectively. Of the 26,683 cases, 11,483 (43.0%) individuals were unvaccinated (2,883 Delta and 8,600 Omicron), and 15,200 (57.0%) individuals were vaccinated with mRNA-1273 (1,431 Delta and 13,769 Omicron; 416 vaccinated with one dose, 12,029 vaccinated with two doses and 2,755 vaccinated with three doses). The flow chart depicting the selection steps is provided (Fig. 1). The distribution of covariates by test outcomes, separated by variant type, is summarized in Table 1 (two-dose and three-dose analyses) and Supplementary Table 1 (one-dose analysis).

**Table 1:** Characteristics of SARS-CoV-2 cases and controls by variant

	Two-dose						Three-dose					
	Delta			Omicron			Delta			Omicron		
	Test positive cases	Test negative controls	*P* value/ASD	Test positive cases	Test negative controls	*P* value/ASD	Test positive cases	Test negative controls	*P* value/ASD	Test positive cases	Test negative controls	*P* value/ASD
	*n* = 4,117	*n* = 8,234		*n* = 19,395	*n* = 38,790		*n* = 3,021	*n* = 6,042		*n* = 11,217	*n* = 22,434	
Age at specimen collection date, years			0.39 / 0.02			<0.01 / 0.04			0.04 / 0.05			<0.01 / 0.07
Mean (s.d.)	42.31 (14.64)	42.60 (14.67)		39.10 (13.77)	39.68 (13.94)		41.81 (14.67)	42.48 (14.58)		40.61 (15.08)	41.65 (15.15)	
Median	41	40		37	38		40	40		38	39	
Q1, Q3	31, 53	31, 53		28, 49	29, 50		31, 52	32, 53		29, 51	30, 52	
Min, max	18, 92	18, 97		18, 93	18, 101		18, 90	18, 98		18, 99	18, 103	
Age at specimen collection date, years, *n* (%)			N/A				N/A		N/A			N/A
18–44	2,458 (59.7%)	4,916 (59.7%)		13,017 (67.1%)	26,034 (67.1%)		1,855 (61.4%)	3,710 (61.4%)		7,211 (64.3%)	14,422 (64.3%)	
45–64	1,339 (32.5%)	2,678 (32.5%)		5,519 (28.5%)	11,038 (28.5%)		933 (30.9%)	1,866 (30.9%)		3,067 (27.3%)	6,134 (27.3%)	
65–74	242 (5.9%)	484 (5.9%)		652 (3.4%)	1,304 (3.4%)		177 (5.9%)	354 (5.9%)		691 (6.2%)	1,382 (6.2%)	
≥75	78 (1.9%)	156 (1.9%)		207 (1.1%)	414 (1.1%)		56 (1.9%)	112 (1.9%)		248 (2.2%)	496 (2.2%)	
Sex, *n* (%)			N/A			N/A			N/A			N/A
Female	2,224 (54.0%)	4,448 (54.0%)		11,124 (57.4%)	22,248 (57.4%)		1,594 (52.8%)	3,188 (52.8%)		6,345 (56.6%)	12,690 (56.6%)	
Male	1,893 (46.0%)	3,786 (46.0%)		8,271 (42.6%)	16,542 (42.6%)		1,427 (47.2%)	2,854 (47.2%)		4,872 (43.4%)	9,744 (43.4%)	
Race/ethnicity, *n* (%)			N/A			N/A			N/A			N/A
Non-Hispanic White	1,575 (38.3%)	3,150 (38.3%)		4,962 (25.6%)	9,924 (25.6%)		1,193 (39.5%)	2,386 (39.5%)		3,240 (28.9%)	6,480 (28.9%)	
Non-Hispanic Black	235 (5.7%)	470 (5.7%)		1,750 (9.0%)	3,500 (9.0%)		186 (6.2%)	372 (6.2%)		1,151 (10.3%)	2,302 (10.3%)	
Hispanic	1,812 (44.0%)	3,624 (44.0%)		9,482 (48.9%)	18,964 (48.9%)		1,279 (42.3%)	2,558 (42.3%)		5,127 (45.7%)	10,254 (45.7%)	
Non-Hispanic Asian	180 (4.4%)	360 (4.4%)		1,540 (7.9%)	3,080 (7.9%)		120 (4.0%)	240 (4.0%)		809 (7.2%)	1,618 (7.2%)	
Other/Unknown	315 (7.7%)	630 (7.7%)		1,661 (8.6%)	3,322 (8.6%)		243 (8.0%)	486 (8.0%)		890 (7.9%)	1,780 (7.9%)	
Body mass index^b^, *n* (%)			<0.01 / 0.14			<0.01 / 0.08			<0.01 / 0.18			<0.01 / 0.11
<18.5	26 (0.6%)	82 (1.0%)		180 (0.9%)	430 (1.1%)		18 (0.6%)	64 (1.1%)		129 (1.2%)	250 (1.1%)	
18.5 – <25	744 (18.1%)	1,672 (20.3%)		3,854 (19.9%)	8,076 (20.8%)		567 (18.8%)	1355 (22.4%)		2,312 (20.6%)	4,829 (21.5%)	
25 – <30	1,102 (26.8%)	2,250 (27.3%)		5,130 (26.5%)	10,513 (27.1%)		784 (26.0%)	1,675 (27.7%)		3,106 (27.7%)	6,186 (27.6%)	
30 – <35	838 (20.4%)	1,635 (19.9%)		3,733 (19.2%)	7,606 (19.6%)		599 (19.8%)	1,152 (19.1%)		2,124 (18.9%)	4,306 (19.2%)	
35 – <40	411 (10.0%)	860 (10.4%)		1,938 (10.0%)	4,019 (10.4%)		304 (10.1%)	586 (9.7%)		1,054 (9.4%)	2,350 (10.5%)	
40 – <45	168 (4.1%)	387 (4.7%)		914 (4.7%)	1,834 (4.7%)		117 (3.9%)	248 (4.1%)		477 (4.3%)	1,066 (4.8%)	
≥45	106 (2.6%)	265 (3.2%)		601 (3.1%)	1,255 (3.2%)		67 (2.2%)	173 (2.9%)		277 (2.5%)	715 (3.2%)	
Unknown	722 (17.5%)	1,083 (13.2%)		3,045 (15.7%)	5,057 (13.0%)		565 (18.7%)	789 (13.1%)		1,738 (15.5%)	2,732 (12.2%)	
Smoking^b^, *n* (%)			<0.01 / 0.12			<0.01 / 0.08			<0.01 / 0.16			<0.01 / 0.10
No	2,855 (69.3%)	5,942 (72.2%)		14,239 (73.4%)	28,750 (74.1%)		2,037 (67.4%)	4,374 (72.4%)		8,172 (72.9%)	16,622 (74.1%)	
Yes	672 (16.3%)	1,425 (17.3%)		2,709 (14.0%)	6,018 (15.5%)		510 (16.9%)	1,033 (17.1%)		1,647 (14.7%)	3,658 (16.3%)	
Unknown	590 (14.3%)	867 (10.5%)		2,447 (12.6%)	4,022 (10.4%)		474 (15.7%)	635 (10.5%)		1,398 (12.5%)	2,154 (9.6%)	
Charlson comorbidity score^a^, *n* (%)			<0.01 / 0.12			<0.01 / 0.11			<0.01 / 0.12			<0.01 / 0.12
0	3,324 (80.7%)	6,321 (76.8%)		16,149 (83.3%)	30,856 (79.5%)		2,471 (81.8%)	4,689 (77.6%)		9,084 (81.0%)	17,074 (76.1%)	
1	480 (11.7%)	1,007 (12.2%)		2,172 (11.2%)	4,799 (12.4%)		337 (11.2%)	731 (12.1%)		1,254 (11.2%)	3,023 (13.5%)	
≥2	313 (7.6%)	906 (11.0%)		1,074 (5.5%)	3,135 (8.1%)		213 (7.1%)	622 (10.3%)		879 (7.8%)	2,337 (10.4%)	
Frailty index^a^, *n* (%)			<0.01 / 0.17			<0.01 / 0.12			<0.01 / 0.19			<0.01 / 0.14
Quartile 1	988 (24.0%)	1,925 (23.4%)		4,926 (25.4%)	9,615 (24.8%)		722 (23.9%)	1,451 (24.0%)		2,729 (24.3%)	5,490 (24.5%)	
Quartile 2	1,249 (30.3%)	2,013 (24.4%)		5,284 (27.2%)	9,158 (23.6%)		935 (31.0%)	1,418 (23.5%)		3,234 (28.8%)	5,371 (23.9%)	
Quartile 3	1,014 (24.6%)	2,071 (25.2%)		4,952 (25.5%)	9,700 (25.0%)		735 (24.3%)	1,537 (25.4%)		2,831 (25.2%)	5,584 (24.9%)	
Quartile 4 (most frail)	866 (21.0%)	2,225 (27.0%)		4,233 (21.8%)	10,317 (26.6%)		629 (20.8%)	1,636 (27.1%)		2,423 (21.6%)	5,989 (26.7%)	
Chronic diseases^a^, *n* (%)												
Kidney disease	78 (1.9%)	252 (3.1%)	<0.01 / 0.08	205 (1.1%)	823 (2.1%)	<0.01 / 0.09	56 (1.9%)	175 (2.9%)	<0.01 / 0.07	227 (2.0%)	613 (2.7%)	<0.01 / 0.05
Heart disease	52 (1.3%)	180 (2.2%)	<0.01 / 0.07	160 (0.8%)	612 (1.6%)	<0.01 / 0.07	41 (1.4%)	119 (2.0%)	0.04 / 0.05	140 (1.2%)	386 (1.7%)	<0.01 / 0.04
Lung disease	284 (6.9%)	713 (8.7%)	<0.01 / 0.07	1,217 (6.3%)	3,148 (8.1%)	<0.01 / 0.07	205 (6.8%)	530 (8.8%)	<0.01 / 0.07	774 (6.9%)	2,053 (9.2%)	<0.01 / 0.08
Liver disease	111 (2.7%)	311 (3.8%)	<0.01 / 0.06	461 (2.4%)	1,161 (3.0%)	<0.01 / 0.04	74 (2.4%)	195 (3.2%)	0.04 / 0.05	271 (2.4%)	730 (3.3%)	<0.01 / 0.05
Diabetes	310 (7.5%)	761 (9.2%)	<0.01 / 0.06	1,318 (6.8%)	3,112 (8.0%)	<0.01 / 0.05	190 (6.3%)	492 (8.1%)	<0.01 / 0.07	831 (7.4%)	2,152 (9.6%)	<0.01 / 0.08
Immunocompromised, *n* (%)	67 (1.6%)	267 (3.2%)	<0.01 / 0.10	332 (1.7%)	1,068 (2.8%)	<0.01 / 0.07	46 (1.5%)	245 (4.1%)	<0.01 / 0.15	274 (2.4%)	832 (3.7%)	<0.01 / 0.07
HIV/AIDS	3	27		37	82		2	28		37	99	
Leukemia/lymphoma, congenital and other immunodeficiencies, asplenia/hyposplenia	28	86		102	325		17	84		94	255	
Hematopoietic stem cell transplantation/organ transplant	6	22		15	75		4	25		29	84	
Immunosuppressant medications	38	168		212	736		29	158		173	560	
Autoimmune conditions^a^, *n* (%)	94 (2.3%)	221 (2.7%)	0.18 / 0.03	351 (1.8%)	841 (2.2%)	<0.01 / 0.03	66 (2.2%)	183 (3.0%)	0.02 / 0.05	253 (2.3%)	659 (2.9%)	<0.01 / 0.04
Rheumatoid arthritis	29	107		125	350		19	77		100	282	
Inflammatory bowel disease	22	52		77	206		17	52		63	157	
Psoriasis and psoriatic arthritis	37	56		129	241		25	5		74	197	
Multiple sclerosis	7	13		23	57		5	9		19	34	
Systemic lupus erythematosus	5	21		32	98		3	25		33	88	
Pregnant at specimen collection date, *n* (%)	70 (1.7%)	244 (3.0%)	<0.01 / 0.08	343 (1.8%)	1213 (3.1%)	<0.01 / 0.09	58 (1.9%)	187 (3.1%)	<0.01 / 0.08	224 (2.0%)	691 (3.1%)	<0.01 / 0.07
1st trimester	20	29		68	175		16	32		40	78	
2nd trimester	22	67		133	308		20	51		80	149	
3rd trimester	28	148		142	730		22	104		104	464	
History of COVID-19^c^, *n* (%)	103 (2.5%)	1,637 (19.9%)	<0.01 / 0.57	2,639 (13.6%)	7,866 (20.3%)	<0.01 / 0.18	92 (3.0%)	1200 (19.9%)	<0.01 / 0.55	1,731 (15.4%)	4,062 (18.1%)	<0.01 / 0.07
History of SARS-CoV-2 molecular test^c^, *n* (%)	2,722 (66.1%)	6,456 (78.4%)	<0.01 / 0.28	13,994 (72.2%)	28,950 (74.6%)	<0.01 / 0.06	1,954 (64.7%)	4,824 (79.8%)	<0.01 / 0.34	8,199 (73.1%)	16,894 (75.3%)	<0.01 / 0.05
Number of outpatient and virtual visits^a^, *n* (%)			<0.01 / 0.31			<0.01 / 0.19			<0.01 / 0.38			<0.01 / 0.27
0	501 (12.2%)	571 (6.9%)		1,624 (8.4%)	2,510 (6.5%)		453 (15.0%)	491 (8.1%)		1,202 (10.7%)	1,434 (6.4%)	
1–4	1,450 (35.2%)	2,220 (27.0%)		6,680 (34.4%)	11,329 (29.2%)		1,121 (37.1%)	1,630 (27.0%)		3,774 (33.6%)	5,884 (26.2%)	
5–10	1,109 (26.9%)	2,401 (29.2%)		5,915 (30.5%)	11,529 (29.7%)		731 (24.2%)	1,656 (27.4%)		3,060 (27.3%)	6,420 (28.6%)	
≥11	1,057 (25.7%)	3,042 (36.9%)		5176 (26.7%)	13422 (34.6%)		716 (23.7%)	2,265 (37.5%)		3,181 (28.4%)	8,696 (38.8%)	
Number of emergency department visits^a^, *n* (%)			<0.01 / 0.16			<0.01 / 0.13			<0.01 / 0.13			<0.01 / 0.09
0	3,503 (85.1%)	6,528 (79.3%)		16,378 (84.4%)	31,250 (80.6%)		2,580 (85.4%)	4,878 (80.7%)		9,362 (83.5%)	18,132 (80.8%)	
1	443 (10.8%)	1,139 (13.8%)		2,270 (11.7%)	5,066 (13.1%)		316 (10.5%)	817 (13.5%)		1,366 (12.2%)	2,903 (12.9%)	
≥2	171 (4.2%)	567 (6.9%)		747 (3.9%)	2,474 (6.4%)		125 (4.1%)	347 (5.7%)		489 (4.4%)	1,399 (6.2%)	
Number of hospitalizations^a^, *n* (%)			<0.01 / 0.09			<0.01 / 0.10			0.01 / 0.07			<0.01 / 0.08
0	3,923 (95.3%)	7,697 (93.5%)		18,675 (96.3%)	36,624 (94.4%)		2,873 (95.1%)	5,670 (93.8%)		10,743 (95.8%)	21,177 (94.4%)	
1	162 (3.9%)	411 (5.0%)		630 (3.2%)	1,707 (4.4%)		123 (4.1%)	280 (4.6%)		416 (3.7%)	1,005 (4.5%)	
≥2	32 (0.8%)	126 (1.5%)		90 (0.5%)	459 (1.2%)		25 (0.8%)	92 (1.5%)		58 (0.5%)	252 (1.1%)	
Preventive care^a^, *n* (%)	2,186 (53.1%)	4,909 (59.6%)	<0.01 / 0.13	10,773 (55.5%)	23,352 (60.2%)	<0.01 / 0.09	1,450 (48.0%)	3,660 (60.6%)	<0.01 / 0.25	6,114 (54.5%)	14,617 (65.2%)	<0.01 / 0.22
Medicaid, *n* (%)	391 (9.5%)	844 (10.3%)	0.19 / 0.03	1,897 (9.8%)	4,461 (11.5%)	<0.01 / 0.06	310 (10.3%)	581 (9.6%)	0.33 / 0.02	1,187 (10.6%)	2,425 (10.8%)	0.53 / 0.01
Neighborhood median household income, *n* (%)			0.05 / 0.06			<0.01 / 0.05			<0.01 / 0.09			0.03 / 0.04
<$40,000	179 (4.3%)	402 (4.9%)		812 (4.2%)	1,902 (4.9%)		129 (4.3%)	243 (4.0%)		458 (4.1%)	1,070 (4.8%)	
$40,000–$59,999	712 (17.3%)	1,580 (19.2%)		3,856 (19.9%)	8,082 (20.8%)		494 (16.4%)	1171 (19.4%)		2,175 (19.4%)	4,392 (19.6%)	
$60,000–$79,999	1,097 (26.6%)	2,121 (25.8%)		5,146 (26.5%)	9,948 (25.6%)		817 (27.0%)	1,483 (24.5%)		2,931 (26.1%)	5,740 (25.6%)	
$80,000+	2,126 (51.6%)	4,123 (50.1%)		9,563 (49.3%)	18,817 (48.5%)		1,579 (52.3%)	3,141 (52.0%)		5,636 (50.2%)	11,211 (50.0%)	
Unknown	3 (0.1%)	8 (0.1%)		18 (0.1%)	41 (0.1%)		2 (0.1%)	4 (0.1%)		17 (0.2%)	21 (0.1%)	
KPSC physician/employee, *n* (%)	129 (3.1%)	609 (7.4%)	<0.01 / 0.19	806 (4.2%)	1759 (4.5%)	0.04 / 0.02	85 (2.8%)	558 (9.2%)	<0.01 / 0.27	480 (4.3%)	1,176 (5.2%)	<0.01 / 0.05
Specimen type, *n* (%)			<0.01 / 0.39			<0.01 / 0.21			<0.01 / 0.47			<0.01 / 0.17
Nasopharyngeal/oropharyngeal swab	3,627 (88.1%)	5,990 (72.7%)		17,162 (88.5%)	31,379 (80.9%)		2,607 (86.3%)	4,042 (66.9%)		9,513 (84.8%)	17,523 (78.1%)	
Saliva	490 (11.9%)	2,244 (27.3%)		2,233 (11.5%)	7,411 (19.1%)		414 (13.7%)	2,000 (33.1%)		1,704 (15.2%)	4,911 (21.9%)	

^a^Defined in the 1 year before specimen collection date

^b^Defined in the 2 years before specimen collection date

^c^Defined on the basis of all available medical records from 1 March 2020 to specimen collection date

Medical center area is not shown. There were differences in the distribution of the vaccinated and unvaccinated individuals across the 19 medical center areas.

ASD, absolute standardized difference; N/A, not applicable.

Omicron cases more frequently had a history of COVID-19 (SARS-CoV-2 infection) than Delta cases. In the two-dose and three-dose analyses, 13.6% and 15.4% of Omicron cases in the two-dose and three-dose analyses, respectively, had a history of COVID-19 (SARS-CoV-2 infection) versus 2.5% and 3.0% of Delta cases (Table 1).

Table 2 shows VE against Delta and Omicron infection or hospitalization. Overall, the one-dose VE was 56.7% (95% confidence interval (CI), 40.7–68.4%) and 20.4% (9.5–30.0%) against Delta and Omicron infection, respectively.

**Table 2:** VE of mRNA-1273 against infection and hospitalization with Delta or Omicron variants

		SARS-CoV-2 test positive		SARS-CoV-2 test negative		VE (95% CI)^a^	
	Variant	Vaccinated (%)	Unvaccinated (%)	Vaccinated (%)	Unvaccinated (%)	Unadjusted	Adjusted
**Infection**^b,c^							
One-dose	Delta	59 (2.0%)	2,883 (98.0%)	218 (3.7%)	5,666 (96.3%)	47.0% (29.0%, 60.4%)	56.7% (40.7%, 68.4%)
	Omicron	357 (4.0%)	8,590 (96.0%)	843 (4.7%)	17,051 (95.3%)	15.8% (4.5%, 25.8%)	20.4% (9.5%, 30.0%)
Two-dose	Delta	1,234 (30.0%)	2,883 (70.0%)	4,031 (49.0%)	4,203 (51.0%)	57.0% (53.3%, 60.4%)	63.6% (59.9%, 66.9%)
	14–90 days	21 (0.7%)	2,883 (99.3%)	151 (3.5%)	4,203 (96.5%)	79.7% (67.9%, 87.2%)	80.2% (68.2%, 87.7%)
	91–180 days	87 (2.9%)	2,883 (97.1%)	342 (7.5%)	4,203 (92.5%)	62.9% (52.9%, 70.8%)	68.9% (60.1%, 75.8%)
	181–270 days	824 (22.2%)	2,883 (77.8%)	2,663 (38.8%)	4,203 (61.2%)	54.9% (50.6%, 58.8%)	63.7% (59.8%, 67.2%)
	>270 days	302 (9.5%)	2,883 (90.5%)	875 (17.2%)	4,203 (82.8%)	49.7% (42.2%, 56.2%)	61.3% (55.0%, 66.7%)
	Omicron	10,795 (55.7%)	8,600 (44.3%)	22,679 (58.5%)	16,111 (41.5%)	11.2% (8.0%, 14.3%)	13.9% (10.5%, 17.1%)
	14–90 days	245 (2.8%)	8,600 (97.2%)	836 (4.9%)	16,111 (95.1%)	45.1% (36.5%, 52.5%)	44.0% (35.1%, 51.6%)
	91–180 days	783 (8.3%)	8,600 (91.7%)	1,867 (10.4%)	16,111 (89.6%)	21.4% (14.3%, 28.0%)	23.5% (16.4%, 30.0%)
	181–270 days	7,015 (44.9%)	8,600 (55.1%)	14,759 (47.8%)	16,111 (52.2%)	11.0% (7.5%, 14.3%)	13.8% (10.2%, 17.3%)
	>270 days	2,752 (24.2%)	8,600 (75.8%)	5217 (24.5%)	16,111 (75.5%)	1.2% (−4.0%, 6.3%)	5.9% (0.4%, 11.0%)
Three-dose	Delta	138 (4.6%)	2,883 (95.4%)	1,836 (30.4%)	4,206 (69.6%)	93.6% (92.0%, 95.0%)	94.5% (92.9%, 95.7%)
	14–60 days	112 (3.7%)	2,883 (96.3%)	1,658 (28.3%)	4,206 (71.7%)	90.1% (88.0%, 91.9%)	93.7% (92.2%, 94.9%)
	>60 days	26 (0.9%)	2,883 (99.1%)	178 (4.1%)	4,206 (95.9%)	78.7% (67.8%, 85.9%)	86.0% (78.1%, 91.1%)
	Omicron	2,617 (23.3%)	8,600 (76.7%)	10,203 (45.5%)	12,231 (54.5%)	71.5% (69.7%, 73.1%)	70.0% (68.0%, 71.9%)
	14–60 days	2,127 (19.8%)	8,600 (80.2%)	9,121 (42.7%)	12,231 (57.3%)	66.8% (65.0%, 68.6%)	71.6% (69.7%, 73.4%)
	>60 days	490 (5.4%)	8,600 (94.6%)	1,082 (8.1%)	12,231 (91.9%)	35.6% (28.1%, 42.3%)	47.4% (40.5%, 53.5%)
Three-dose excluding immunocompromised patients	Delta	124 (4.2%)	2,851 (95.8%)	1,708 (29.5%)	4,089 (70.5%)	89.6% (87.4%, 91.4%)	93.7% (92.2%, 94.9%)
	14–60 days	104 (3.5%)	2,851 (96.5%)	1,580 (27.9%)	4,089 (72.1%)	90.6% (88.4%, 92.3%)	94.2% (92.7%, 95.3%)
	>60 days	20 (0.7%)	2,851 (99.3%)	128 (3.0%)	4,089 (97.0%)	77.6% (64.0%, 86.0%)	88.1% (80.2%, 92.9%)
	Omicron	2,464 (22.5%)	8,479 (77.5%)	9,677 (44.8%)	11,925 (55.2%)	64.2% (62.3%, 66.0%)	70.5% (68.6%, 72.4%)
	14–60 days	2,059 (19.5%)	8,479 (80.5%)	8,803 (42.5%)	11,925 (57.5%)	67.1% (65.2%, 68.9%)	72.1% (70.2%, 73.9%)
	>60 days	405 (4.6%)	8,479 (95.4%)	874 (6.8%)	11,925 (93.2%)	34.8% (26.4%, 42.3%)	51.2% (44.2%, 57.3%)
**Hospitalization**^b,d^							
One-dose	Delta^e^	1 (1.3%)	79 (98.8%)	10 (6.3%)	150 (93.8%)	82.2% (−31.4%, 97.8%)	71.2% (−68.7%, 97.4%)
	Omicron	0 (0.0%)	14 (100.0%)	2 (7.1%)	26 (92.9%)	100.0% (N/A)	N/A
Two-dose	Delta^e^	4 (4.8%)	79 (95.2%)	94 (56.6%)	72 (43.4%)	95.9% (86.9%, 98.7%)	99.0% (93.3%, 99.9%)
	Omicron^f^	7 (33.3%)	14 (66.7%)	28 (66.7%)	14 (33.3%)	81.1% (29.8%, 94.9%)	84.5% (23.0%, 96.9%)
Three-dose	Delta^e^	1 (1.3%)	79 (98.8%)	69 (43.1%)	91 (56.9%)	98.3% (87.7%, 99.8%)	99.7% (96.5%, 100.0%)
	Omicron^g^	4 (22.2%)	14 (77.8%)	26 (72.2%)	10 (27.8%)	89.0% (58.5%, 97.1%)	99.2% (76.3%, 100.0%)

^a^When the OR or its 95% CI was >1, the VE or its 95% CI was transformed as −(1 − [1/adjusted OR]) × 100 (ref. ^24^).

^b^Models for time since vaccination analyses and three-dose hospitalization analyses are unconditional logistic models with adjustment for matching variables.

^c^Model adjusted for core variables: history of SARS-CoV-2 molecular test; preventive care; number of outpatient and virtual visits; Charlson comorbidity score; obesity (yes/no/unknown); frailty index; specimen type; immunocompromised status and history of COVID-19.

^d^Model adjusted for core variables: history of SARS-CoV-2 molecular test; preventive care; Charlson comorbidity score; obesity (yes/no/unknown); immunocompromised status and history of COVID-19.

^e^Immunocompromised status was removed from the list of core variables owing to lack of model convergence.

^f^Obesity was removed from the list of core variables owing to lack of model convergence.

^g^Obesity and history of COVID-19 were removed from the list of core variables owing to lack of model convergence.

**Fig. 2:**
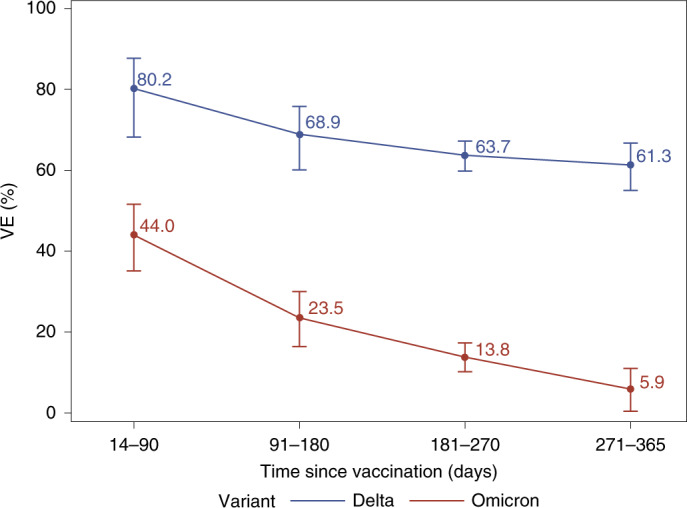
VE of two doses of mRNA-1273 against Omicron and Delta variants by time since vaccination (*n* = 70,536 individuals). Waning effectiveness of two doses of mRNA-1273 vaccine against Omicron infection (red line) and Delta infection (blue line) within 365 days after receipt of second dose. Data are presented as VE ± 95% CI.

**Fig. 3:**
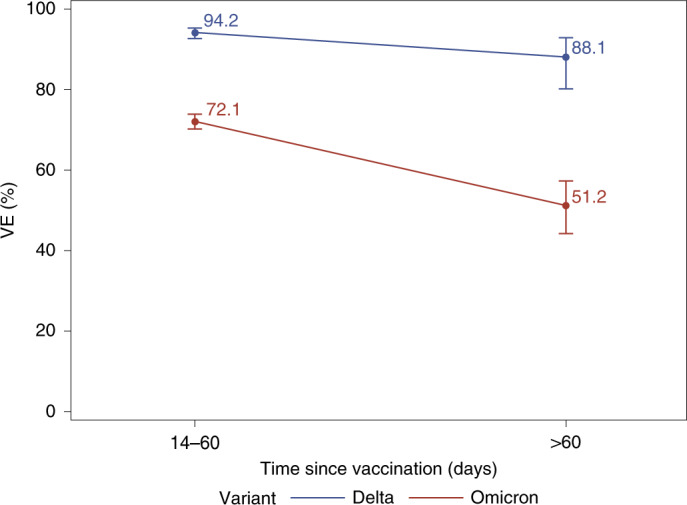
VE of three doses of mRNA-1273 against Omicron and Delta variants by time since vaccination among immunocompetent population (*n* = 42,714 individuals). Effectiveness of three doses of mRNA-1273 vaccine against Delta infection (blue line) and Omicron infection (red line), comparing effectiveness by time since third dose (14–60 days or >60 days). Data are presented as VE ± 95% CI.

In analyses of two-dose VE against Delta infection by time since receipt of dose 2, VE at 14–90 days was 80.2% (68.2–87.7%) and subsequently declined, with VE of 68.9% (60.1–75.8%) at 91–180 days, 63.7% (59.8–67.2%) at 181–270 days and 61.3% (55.0–66.7%) at >270 days (Table 2 and Fig. 2). In comparison, the two-dose VE against Omicron infection was 44.0% (35.1–51.6%) at 14–90 days and declined quickly to 23.5% (16.4–30.0%) at 91–180 days, 13.8% (10.2–17.3%) at 181–270 days and 5.9% (0.4–11.0%) at >270 days. The three-dose VE against Delta infection was 93.7% (92.2–94.9%) at 14–60 days and 86.0% (78.1–91.1%) at >60 days. However, the three-dose VE against Omicron infection was 71.6% (69.7–73.4%) at 14–60 days and 47.4% (40.5–53.5%) at >60 days. These estimates were similar in analyses that excluded individuals who were immunocompromised, except that the three-dose VE against Omicron infection increased to 51.2% (44.2–57.3%) among immunocompetent individuals at >60 days (Table 2 and Fig. 3).

The VE of two and three doses against hospitalization with Delta were both ≥99%, whereas they were 84.5% (23.0–96.9%) and 99.2% (76.3–100.0%), respectively, against hospitalization with Omicron (Table 2). Notably, all four individuals hospitalized with Omicron despite receipt of three mRNA-1273 doses were older than 60 years of age with chronic diseases, and one was also immunocompromised.

Table 3 presents the three-dose VE against infection by subgroups. The three-dose VE against Delta infection was more than 93% across age, sex and race/ethnicity groups but lower in the immunocompromised population (70.6% (31.0–87.5%), *P* value for interaction <0.001). The three-dose VE against Omicron infection was 70.9% (68.9–72.9%) in those aged <65 years and 64.3% (55.0–71.7%) in those aged ≥65 years and only 29.4% (0.3–50.0%) in the immunocompromised population compared to 70.5% (68.6–72.4%) in the immunocompetent population (*P* value for interaction <0.001). The three-dose VE against Omicron infection among those who had no history of COVID-19 was 70.1% (68.0–72.1%) in those aged <65 years and 64.5% (54.9–72.1%) in those aged ≥65 years.

**Table 3:** VE of three doses of mRNA-1273 against infection with Delta or Omicron variants by subgroup

	SARS-CoV-2 test positive		SARS-CoV-2 test negative		VE (95% CI)		
Variant^a,b^	Vaccinated (%)	Unvaccinated (%)	Vaccinated (%)	Unvaccinated (%)	Unadjusted	Adjusted	*P* value for interaction
Delta							
Age at specimen collection date							0.3742
<65	94 (3.4%)	2,694 (96.6%)	1,470 (26.4%)	4,106 (73.6%)	93.3% (91.3%, 94.8%)	94.3% (92.5%, 95.7%)	
≥65	44 (18.9%)	189 (81.1%)	366 (78.5%)	100 (21.5%)	95.0% (91.1%, 97.1%)	96.0% (92.3%, 97.9%)	
Sex							0.8922
Female	75 (4.7%)	1,519 (95.3%)	969 (30.4%)	2,219 (69.6%)	93.2% (90.7%, 95.0%)	94.4% (92.2%, 96.0%)	
Male	63 (4.4%)	1,364 (95.6%)	867 (30.4%)	1,987 (69.6%)	94.2% (91.7%, 95.9%)	94.6% (92.0%, 96.3%)	
Race/ethnicity							0.1993
Hispanic	39 (3.0%)	1,240 (97.0%)	577 (22.6%)	1,981 (77.4%)	92.4% (88.7%, 94.8%)	93.1% (89.4%, 95.5%)	
Non-Hispanic and others	99 (5.7%)	1,643 (94.3%)	1,259 (36.1%)	2,225 (63.9%)	94.2% (92.2%, 95.7%)	95.1% (93.2%, 96.4%)	
Immunocompromised status							0.0002
Yes^c^	14 (30.4%)	32 (69.6%)	128 (52.2%)	117 (47.8%)	60.0% (21.4%, 79.7%)	70.6% (31.0%, 87.5%)	
No	124 (4.2%)	2,851 (95.8%)	1,708 (29.5%)	4,089 (70.5%)	89.6% (87.4%, 91.4%)	93.7% (92.2%, 94.9%)	
Omicron							
Age at specimen collection date							0.0969
<65	1,943 (18.9%)	8,335 (81.1%)	8,573 (41.7%)	11,983 (58.3%)	72.2% (70.4%, 73.9%)	70.9% (68.9%, 72.9%)	
≥65	674 (71.8%)	265 (28.2%)	1,630 (86.8%)	248 (13.2%)	61.7% (53.2%, 68.6%)	64.3% (55.0%, 71.7%)	
Sex							0.9159
Female	1,529 (24.1%)	4,816 (75.9%)	5,862 (46.2%)	6,828 (53.8%)	70.4% (67.9%, 72.6%)	70.0% (67.4%, 72.4%)	
Male	1,088 (22.3%)	3,784 (77.7%)	4,341 (44.6%)	5,403 (55.4%)	72.9% (70.3%, 75.3%)	70.0% (66.6%, 72.9%)	
Race/ethnicity							0.0866
Hispanic	970 (18.9%)	4,157 (81.1%)	3,976 (38.8%)	6,278 (61.2%)	69.6% (66.7%, 72.2%)	68.0% (64.6%, 71.0%)	
Non-Hispanic and others	1,647 (27.0%)	4,443 (73.0%)	6,227 (51.1%)	5,953 (48.9%)	72.8% (70.5%, 74.9%)	71.4% (68.8%, 73.8%)	
Immunocompromised status							<.0001
Yes	153 (55.8%)	121 (44.2%)	526 (63.2%)	306 (36.8%)	26.4% (3.0%, 44.2%)	29.4% (0.3%, 50.0%)	
No	2,464 (22.5%)	8,479 (77.5%)	9,677 (44.8%)	11,925 (55.2%)	64.2% (62.3%, 66.0%)	70.5% (68.6%, 72.4%)	

^a^Models for immunocompromised status subgroup analyses are unconditional logistic models with adjustment for matching variables.

^b^Model adjusted for core variables: history of SARS-CoV-2 molecular test; preventive care; number of outpatient and virtual visits; Charlson comorbidity score; obesity (yes/no/unknown); frailty index; specimen type; immunocompromised status and history of COVID-19

^c^Number of outpatient and virtual visits was removed from the list of core variables owing to lack of model convergence.

## Discussion

We evaluated the effectiveness of mRNA-1273 against the highly mutated Omicron variant in a socio-demographically diverse population in a real-world setting. Between 6 December 2021 and 31 December 2021, the rapidly increasing proportion of Omicron-positive specimens indicated unprecedented transmissibility and raised concerns over protection conferred by currently authorized or licensed COVID-19 vaccines. Our study demonstrates that, although VE of two doses of mRNA-1273 against Delta infection is high and wanes slowly, consistent with our previous findings^6,14^, the two-dose VE against Omicron infection is inadequate, providing only modest protection of 44.0% within 3 months of vaccination and diminishing quickly thereafter. In addition, although the three-dose VE against Delta infection is high and durable, that against Omicron is lower. Nevertheless, the average point estimate (>50%) and lower bound of the 95% CI (>30%) still meet the US FDA criteria for emergency use authorization for COVID-19 vaccines^15^. Also, this level of VE is similar to the two-dose vaccine efficacy against asymptomatic infection observed in the phase 3 clinical trial (63.0% (56.6–68.5%))^16^. The VE of three doses of mRNA-1273 against Omicron infection is poor among individuals who are immunocompromised. Although two-dose VE against hospitalization with Omicron is lower compared to that with Delta, three-dose VE is nearly 100% against hospitalization with either variant. Although additional study is needed, these findings suggest that third (booster) doses may be needed <6 months after dose 2 in immunocompetent individuals and that three doses may be inadequate to protect against Omicron infection in individuals who are immunocompromised. Furthermore, the data indicate a potential need for periodic adjustment of vaccines to target circulating variants that have evolved to escape current vaccine-induced immunity.

Although there are limited prior data on VE of two or three doses of mRNA-1273 vaccine against infection or hospitalization with Omicron, a preliminary analysis from Denmark found an initial VE of two doses of mRNA-1273 against Omicron infection of 36.7% that waned quickly^17^, which was similar to our findings. An early report by Andrews et al.^18^ found waning of two-dose protection with an initial VE of two doses of BNT162b2 against symptomatic Omicron infection of 65.5% (63.9–67.0%) two to four weeks after the second dose, dropping to 15.4% (14.2–16.6%) after 15 to 19 weeks and further to 8.8% (7.0–10.5%) after 25 or more weeks. After a BNT162b2 booster dose, the VE increased to 67.2% (66.5–67.8%) from two to four weeks before declining to 45.7% (44.7–46.7%) after 10 or more weeks. Collie et al.^19^ found that the VE of two doses of BNT162b2 against hospitalization during a proxy Omicron period was 70% at least 14 days after receipt of dose 2. In England, after a primary course of BNT162b2 vaccine, VE against Omicron infection was initially 70% after a BNT162b2 booster, dropping to 45% after ≥10 weeks, but stayed around 70–75% for up to 9 weeks after an mRNA-1273 booster^12^.

A growing number of reports indicate that Omicron-associated COVID-19 disease is less severe than Delta-associated COVID-19 disease, resulting in a lower risk of hospitalization^1,20^. This might reflect increased replication of Omicron in the upper versus lower respiratory tract, which could also contribute to more efficient transmission, resulting in increased absolute^21^ numbers of hospitalizations. Booster vaccination has the potential to decrease hospital burden and improve clinical outcomes^22^. Although the sample size and follow-up period were not sufficient in our study or other studies to assess potential waning VE against hospitalization with Omicron, our results of waning VE against Omicron infection after dose 3 of mRNA-1273 underscores the importance of monitoring VE against hospitalization with Omicron infection.

This study was representative of a large and diverse racial, ethnic and socioeconomic population in Southern California. It provides data complementing recent reports of the effectiveness of other COVID-19 vaccines against Omicron infection and has several strengths and limitations^14,23^. First, the results of our test-negative case–control study may not be generalizable to people who are not tested, including those with milder symptoms who might not pursue testing. Although there is a variety of reasons for testing that could introduce biases, we attempted to reduce these biases by accounting for sociodemographic characteristics, prior healthcare use, SARS-CoV-2 testing and comorbidities in the models. Although potential residual confounding or detection bias could remain, these were not likely to affect the conclusions of the study. Although misclassification of disease status was a potential source of bias, we used a highly specific and sensitive RT–PCR test that likely minimized misclassification and enabled us to monitor variant proportions through WGS and SGTF analysis. Similarly, misclassification of vaccination status was possible but likely minimal and non-differential with respect to COVID-19 disease status. KPSC electronic vaccination records that captured all vaccine administrations given at KPSC were updated daily with vaccine administration data from the California Immunization Registry, to which all facilities are required by law to report COVID-19 vaccine administrations within 24 hours. Second, we considered all SGTF specimens as Omicron, as our validation samples using WGS showed high agreement. Our rate of SGTF closely mirrored regional trends in Omicron emergence from the US Centers for Disease Control and Prevention^13^. Delta accounted for 99% of variants for 4 months before the emergence of Omicron in Southern California in December 2021. Furthermore, during the study interval, Delta and Omicron accounted for more than 99% of variants, and the BA.2 sub-lineage of Omicron was not detected among any of the 1,383 specimens sequenced in this study. Therefore, it is reasonable to posit that all variants exhibiting SGTF were Omicron, whereas those without SGTF were Delta during the study interval. Third, some individuals who were immunocompetent and who received a third dose before the 21 October 2021 Advisory Committee on Immunization Practices recommendation may have received a 100-µg dose rather than a 50-µg booster dose of mRNA-1273. However, we were not able to clearly assess the difference, as dosage information was not available from external vaccination records. Fourth, the number of hospitalized individuals included was too small to draw definitive conclusions regarding VE and durability of three doses in preventing hospitalization. Long-term follow-up is needed to evaluate the durability of both 100-µg and 50-µg booster doses in preventing infection and hospitalization. Fifth, we did not evaluate VE against symptomatic or asymptomatic infection. However, we did find higher VE against COVID-19 hospitalization. Aside from the saliva tests that were collected only in asymptomatic individuals, information on whether infections were symptomatic or asymptomatic was not readily available. For future analyses, we plan to apply a natural language processing algorithm to clinical notes to differentiate symptomatic from asymptomatic SARS-CoV-2 infections. Finally, caution should be taken when interpreting waning VE over time, as some CIs overlapped, and heterogenous composition of the vaccinated population over time could potentially contribute to varying estimates. Among the populations first prioritized for vaccination, the most clinically vulnerable individuals might have contributed to overestimates in waning, although this effect may have been offset to some extent by healthcare workers who were also prioritized for vaccine administration and who likely experienced less waning. Furthermore, early vaccine adopters may have implemented risk-avoidance behaviors that put them at a lower risk of infection.

This study of mRNA-1273 found waning two-dose but high three-dose VE against Delta infection and lower two-dose and three-dose VE against Omicron infection. The two-dose VE against hospitalization with Omicron was lower than with Delta, but the three-dose VE against hospitalization with either variant was high. Protection against Omicron infection waned within 3 months after dose 2, suggesting that a shorter interval between second and booster doses could be beneficial. Lack of protection against Omicron infection in the immunocompromised population underscores the importance of monitoring the effectiveness of the recommended fourth dose (booster) for this population. Continued monitoring of VE against Omicron infection and hospitalization in immunocompetent and immunocompromised individuals and surveillance for the emergence of new SARS-CoV-2 variants are warranted to inform future vaccination strategies.

## Methods

### Study setting

KPSC is an integrated healthcare system that provides care to more than 4.6 million socio-demographically diverse health plan members at 15 hospitals and associated medical offices across Southern California. Comprehensive EHRs used for this study included information on demographics, immunizations, diagnoses, laboratory tests, procedures and pharmacy records. KPSC began administering mRNA-1273 on 18 December 2020. External COVID-19 vaccinations were imported into members’ EHRs daily from external sources, including the California Immunization Registry, Care Everywhere (system on the Epic EHR platform that allows healthcare systems to exchange members’ medical information), claims (for example, retail pharmacies) and self-report by members (with valid documentation).

The study was approved by the KPSC institutional review board. All study staff with access to protected health information were trained in procedures to protect the confidentiality of KPSC member data. A waiver of informed consent was obtained as this is an observational study of authorized and recommended Moderna COVID-19 vaccine administered in the course of routine clinical care. To facilitate the conduct of this study, a waiver was obtained for written Health Insurance Portability and Accountability Act authorization for research involving use of the EHR.

### Laboratory methods

Molecular diagnostic testing for SARS-CoV-2 is available to members who request it for any reason, before procedures and hospital admissions, with and without symptoms. Specimens were primarily collected using nasopharyngeal/oropharyngeal swabs (for symptomatic or asymptomatic individuals) or saliva (for asymptomatic individuals). Specimens were tested using RT–PCR TaqPath COVID-19 High-Throughput Combo Kit (Thermo Fisher Scientific). SGTF was defined as an RT–PCR test in which N and ORF1ab genes were detected (cycle threashold values <37) but S gene was not detected. Specimens with SGTF were considered to be Omicron, whereas positive specimens without SGTF were considered to be Delta.

A random sample of SARS-CoV-2-positive specimens was sent for WGS. Details are described in our previous publication^14^. The SGTF data were compared against WGS results to assess their validity in differentiating variants.

### Study design

A test-negative case–control study design was used in which individuals testing positive for SARS-CoV-2 were defined as cases and individuals testing negative were defined as controls; this design is purported to reduce bias associated with confounding by healthcare-seeking behavior and misclassification of cases^25^. In this study, cases included individuals who tested positive by the RT–PCR TaqPath COVID-19 kit, had specimens collected between 6 December 2021 and 31 December 2021, were aged ≥18 years and had ≥12 months of KPSC membership before the specimen collection date (for accurate ascertainment of exposure status and covariates). Individuals were excluded if they received a COVID-19 vaccine other than mRNA-1273, any dose of mRNA-1273 <14 days before the specimen collection date, two or three doses of mRNA-1273 <24 days apart from previous dose or more than three doses of mRNA-1273 before the specimen collection date. Additional exclusions included a positive SARS-CoV-2 test or COVID-19 diagnosis code ≤90 days before the specimen collection date. COVID-19 hospitalization included hospitalization with a SARS-CoV-2-positive test or hospitalization ≤7 days after a SARS-CoV-2-positive test. COVID-19 hospitalization was confirmed by manual chart review conducted by a physician investigator (B.K.A.) to verify the presence of severe COVID-19 symptoms.

Controls included all individuals who tested negative with specimens collected between 6 December 2021 and 31 December 2021, were aged ≥18 years and had ≥12 months of KPSC membership before the specimen collection date. Randomly sampled controls were 2:1 matched to cases by age (18–44 years, 45–64 years, 65–74 years and ≥75 years), sex, race/ethnicity (non-Hispanic White, non-Hispanic Black, Hispanic, non-Hispanic Asian and Other/Unknown) and specimen collection date. Matching was conducted separately for the one-, two- and three-dose VE analysis. To accommodate variation in real-world practice, analyses did not require dose 3 to be ≥6 months from dose 2, as some members received dose 3 at a shorter interval in this study.

### Exposure

The exposure of interest was one, two or three doses of mRNA-1273. Dose 3 in this analysis included both the 100-µg additional primary dose in individuals who were immunocompromised as well as the 50-µg and 100-ug booster doses in adults.

### Covariates

A comprehensive list of pre-specified potential confounders was identified a priori based on the literature. Demographic and clinical covariates were extracted from EHRs^14^. Variables assessed included socioeconomic status (Medicaid and neighborhood median household income), medical center area, pregnancy status, KPSC physician/employee status, smoking, body mass index (BMI), Charlson comorbidity score, autoimmune conditions, chronic diseases (kidney, heart, lung and liver disease and diabetes), frailty index and immunocompromised status (HIV/AIDS, leukemia/lymphoma, congenital and other immunodeficiencies, asplenia/hyposplenia, hematopoietic stem cell and organ transplant and/or immunosuppressant medications). To account for potential differences in care-seeking or test-seeking behaviors, the following variables were also assessed: healthcare use (virtual, outpatient, emergency department and inpatient encounters), preventive care (other vaccinations, screenings and wellness visits), history of SARS-CoV-2 molecular test performed from 1 March 2020 to specimen collection date (irrespective of result) and history of COVID-19 (positive SARS-CoV-2 molecular test or a COVID-19 diagnosis code) from 1 March 2020 to specimen collection date.

### Statistical analyses

Characteristics of cases and controls for each analysis were compared by using the χ^2^ test or Fisher exact test for categorical variables and two-sample *t-*test or Wilcoxon rank-sum test for continuous variables. Absolute standardized difference was calculated to assess the balance of covariates. The distribution of variant type by vaccination status was tabulated. Conditional logistic regression was used to estimate the adjusted ORs and 95% CIs for vaccination against infection and hospitalization with Delta or Omicron. To harmonize the covariates adjusted across different models so that estimates were comparable, we selected two sets of core variables to be included in all models: one set for infection models and one set for hospitalization models. The selection of core variables was based on prior knowledge, potential associations with infection/hospitalization and model parsimony, allowing us to control for test-seeking and care-seeking behavior, general health status, test type and immunity. For the infection models, the core variables included history of SARS-CoV-2 molecular test, preventive care, number of outpatient and virtual visits, Charlson comorbidity score, obesity (BMI ≥ 30), frailty index, specimen type, immunocompromised status and history of COVID-19. For the hospitalization models, the core variables included history of SARS-CoV-2 molecular test, preventive care, Charlson comorbidity score, obesity (BMI ≥ 30), immunocompromised status and history of COVID-19. Unconditional logistic regression with additional adjustment of matching factors in the model was used when matched sets needed to be broken for certain subgroup analyses or when the conditional model failed to converge. VE (%) was calculated as (1 – adjusted OR) × 100.

We also assessed two-dose and three-dose VE against Delta or Omicron infection by time since receipt of mRNA-1273 dose 2 or 3 (for two-dose VE: 14–90 days, 91–180 days, 181–270 days and >270 days; for three-dose VE: 14–60 days and >60 days). As more immunocompromised individuals might have received dose 3 before the 21 October 2021 Advisory Committee on Immunization Practices booster dose recommendation^26,27^, we conducted a separate analysis that excluded individuals who were immunocompromised to assess durability of protection of three doses in immunocompetent individuals. We also evaluated three-dose VE in select subgroups, including by age (<65 and ≥65 years), sex, race/ethnicity (Hispanic, Non-Hispanic and others) and immunocompromised status (yes or no). The difference between subgroups was tested by including an interaction term for subgroup and vaccination in the model. As VE in individuals with a history of COVID-19 is different from those without^6^, we also evaluated three-dose VE against Omicron infection, stratified by age (<65 years and ≥65 years), among individuals with no history of COVID-19. SAS 9.4 software (SAS Institute) was used for analyses.

### Reporting Summary

Further information on research design is available in the Nature Research Reporting Summary linked to this article.

## Online content

Any methods, additional references, Nature Research reporting summaries, source data, extended data, supplementary information, acknowledgements, peer review information; details of author contributions and competing interests; and statements of data and code availability are available at 10.1038/s41591-022-01753-y.

## Supplementary information

Supplementary Information Supplementary Tables 1–36

Reporting Summary

## Data Availability

Individual-level data reported in this study are not publicly shared. Upon reasonable request and subject to review, KPSC may provide the de-identified aggregate-level data that support the findings of this study. De-identified data may be shared upon approval of an analysis proposal and a signed data access agreement.

## Code availability

Standard epidemiological analyses were conducted using standard commands in SAS 9.4. The commands/code are accessible at https://github.com/YiXLuo/P901-Omicron-Manuscript–Nature-Medicine.
