# Author Correction: Effectiveness of mRNA-1273 against SARS-CoV-2 Omicron and Delta variants

**DOI:** 10.1038/s41591-022-01818-y

**Published:** 2022-04-21

**Authors:** Hung Fu Tseng, Bradley K. Ackerson, Yi Luo, Lina S. Sy, Carla A. Talarico, Yun Tian, Katia J. Bruxvoort, Julia E. Tubert, Ana Florea, Jennifer H. Ku, Gina S. Lee, Soon Kyu Choi, Harpreet S. Takhar, Michael Aragones, Lei Qian

**Affiliations:** 1 Department of Research and Evaluation, Kaiser Permanente Southern California, Pasadena, CA USA; 2 Department of Health Systems Science, Kaiser Permanente Bernard J. Tyson School of Medicine, Pasadena, CA USA; 3 Moderna, Inc, Cambridge, MA USA; 4 Department of Epidemiology, University of Alabama at Birmingham, Birmingham, AL USA

**Keywords:** Viral infection, RNA vaccines

Correction to: *Nature Medicine* 10.1038/s41591-022-01753-y, published online 21 February 2022.

This paper was originally published under a standard Springer Nature license (© The Author(s), under exclusive licence to Springer Nature America, Inc.). It is now available as an open-access paper under a Creative Commons Attribution 4.0 International license, © The Author(s). The error has been corrected in the online version of the article.

