# Supplementary material for: Effectiveness of mRNA-1273 against SARS-CoV-2 Omicron and Delta variants
Source: Nat Med. 2022 Feb 21;28(5):1063–71. doi: 10.1038/s41591-022-01753-y (PMC9117141; doi:10.1038/s41591-022-01753-y)
Supplement: Supplementary file 1 — Supplementary Tables 1–36 [file 41591_2022_1753_MOESM1_ESM.pdf]

nature portfolio

https://doi.org/10.1038/s41591-022-01753-y

Supplementary information

Effectiveness of mRNA-1273 against SARS-CoV-2 Omicron and Delta variants

In the format provided by the authors and unedited

Effectiveness of mRNA-1273 against SARS-CoV-2 omicron and delta variants

Supplementary Tables

Authors: Hung Fu Tseng, PhD,[1,2,*] Bradley K. Ackerson, MD,[1] Yi Luo, PhD,[1] Lina S. Sy, MPH,[1] Carla A. Talarico, PhD,[3] Yun Tian, MS,[1] Katia J. Bruxvoort, PhD,[4] Julia E. Tubert, MPH,[1] Ana Florea, PhD,[1] Jennifer H. Ku, PhD,[1] Gina S. Lee, MPH,[1] Soon Kyu Choi, MPH,[1] Harpreet S. Takhar, MPH,[1] Michael Aragones, MD,[1] Lei Qian, PhD[1]

Affiliations: [1]Department of Research and Evaluation, Kaiser Permanente Southern California, 100 S. Los Robles Ave., Pasadena, CA, 91101, USA; [2]Department of Health Systems Science, Kaiser Permanente Bernard J. Tyson School of Medicine, 98 S. Los Robles Ave., Pasadena, CA, 91101, USA; [3]Moderna Inc., 200 Technology Square, Cambridge, MA, 02139, USA; [4]Department of Epidemiology, University of Alabama at Birmingham, 1665 University Blvd., Birmingham, AL, 35233, USA

Contents

Supplementary Table 1. Characteristics of SARS-CoV-2 cases and controls, by variant (1-dose analysis) 4
Supplementary Table 2. Full model for 1-dose VE against delta variant infection 7
Supplementary Table 3. Full model for 1-dose VE against omicron variant infection 8
Supplementary Table 4. Full model for 2-dose VE against delta variant infection 9
Supplementary Table 5. Full model for 2-dose VE against delta variant infection by time since vaccination........ 10
Supplementary Table 6. Full model for 2-dose VE against omicron variant infection 11
Supplementary Table 7. Full model for 2-dose VE against omicron variant infection by time since vaccination........ 12
Supplementary Table 8. Full model for 3-dose VE against delta variant infection 13
Supplementary Table 9. Full model for 3-dose VE against delta variant infection by time since vaccination........ 14
Supplementary Table 10. Full model for 3-dose VE against omicron variant infection 15
Supplementary Table 11. Full model for 3-dose VE against omicron variant infection by time since vaccination........ 16
Supplementary Table 12. Full model for 3-dose VE against delta variant infection by time since vaccination (excluding immunocompromised population) 17
Supplementary Table 13. Full model for 3-dose VE against omicron variant infection by time since vaccination (excluding immunocompromised population) 18
Supplementary Table 14. Full model for 1-dose VE against delta variant hospitalization 19
Supplementary Table 15. Full model for 2-dose VE against delta variant hospitalization 20
Supplementary Table 16. Full model for 2-dose VE against omicron variant hospitalization 21
Supplementary Table 17. Full model for 3-dose VE against delta variant hospitalization 22
Supplementary Table 18. Full model for 3-dose VE against omicron variant hospitalization 23
Supplementary Table 19. Full model for 3-dose VE against delta variant infection (subgroup of age<65) 24
Supplementary Table 20. Full model for 3-dose VE against delta variant infection (subgroup of age≥65) 25
Supplementary Table 21. Full model for 3-dose VE against delta variant infection (subgroup of female) 26
Supplementary Table 22. Full model for 3-dose VE against delta variant infection (subgroup of male)27
Supplementary Table 23. Full model for 3-dose VE against delta variant infection (subgroup of Hispanic)........ 28
Supplementary Table 24. Full model for 3-dose VE against delta variant infection (subgroup of Non-Hispanic and others)........ 29

Supplementary Table 25. Full model for 3-dose VE against delta variant infection (subgroup of immunocompromised population) 30
Supplementary Table 26. Full model for 3-dose VE against delta variant infection (subgroup of non-immunocompromised) 31
Supplementary Table 27. Full model for 3-dose VE against omicron variant infection (subgroup of age<65) 32
Supplementary Table 28. Full model for 3-dose VE against omicron variant infection (subgroup of age≥65) 33
Supplementary Table 29. Full model for 3-dose VE against omicron variant infection (subgroup of female) 34
Supplementary Table 30. Full model for 3-dose VE against omicron variant infection (subgroup of male) 35
Supplementary Table 31. Full model for 3-dose VE against omicron variant infection (subgroup of Hispanic) 36
Supplementary Table 32. Full model for 3-dose VE against omicron variant infection (subgroup of Non-Hispanic and others) 37
Supplementary Table 33. Full model for 3-dose VE against omicron variant infection (subgroup of immunocompromised population) 38
Supplementary Table 34. Full model for 3-dose VE against omicron variant infection (subgroup of non-immunocompromised) 39
Supplementary Table 35. Full model for 3-dose VE against omicron variant infection among individuals without COVID-19 history, age<65 40
Supplementary Table 36. Full model for 3-dose VE against omicron variant infection among individuals without COVID-19 history, age≥65 41

Supplementary Table 1. Characteristics of SARS-CoV-2 cases and controls, by variant (1-dose analysis)

	Delta			Omicron		
	**Test Positive Cases**	**Test Negative Controls**	**p-value/ASD**	**Test Positive Cases**	**Test Negative Controls**	**p-value/ASD[a]**
	**N=2942**	**N=5884**		**N=8947**	**N=17894**	
Age at specimen collection date, years			0.91 / 0.01			<0.01 / 0.04
mean (sd)	41.24 (14.29)	41.33 (14.36)		37.40 (13.06)	37.92 (13.25)	
median	39	39		35	36	
Q1, Q3	31, 51	31, 51		27, 46	28, 46	
min, max	18, 90	18, 101		18, 93	18, 96	
Age at specimen collection date, years, n (%)			N/A			N/A
18-44	1844 (62.7%)	3688 (62.7%)		6477 (72.4%)	12954 (72.4%)	
45-64	904 (30.7%)	1808 (30.7%)		2188 (24.5%)	4376 (24.5%)	
65-74	153 (5.2%)	306 (5.2%)		238 (2.7%)	476 (2.7%)	
≥75	41 (1.4%)	82 (1.4%)		44 (0.5%)	88 (0.5%)	
Sex, n (%)			N/A			N/A
Female	1550 (52.7%)	3100 (52.7%)		4996 (55.8%)	9992 (55.8%)	
Male	1392 (47.3%)	2784 (47.3%)		3951 (44.2%)	7902 (44.2%)	
Race/Ethnicity, n (%)			N/A			N/A
Non-Hispanic White	1159 (39.4%)	2318 (39.4%)		2526 (28.2%)	5052 (28.2%)	
Non-Hispanic Black	181 (6.2%)	362 (6.2%)		961 (10.7%)	1922 (10.7%)	
Hispanic	1265 (43.0%)	2530 (43.0%)		4319 (48.3%)	8638 (48.3%)	
Non-Hispanic Asian	97 (3.3%)	194 (3.3%)		387 (4.3%)	774 (4.3%)	
Other/Unknown	240 (8.2%)	480 (8.2%)		754 (8.4%)	1508 (8.4%)	
Body mass index[c], n (%)			<0.01 / 0.15			<0.01 / 0.08
<18.5	20 (0.7%)	71 (1.2%)		105 (1.2%)	224 (1.3%)	
18.5 - <25	545 (18.5%)	1273 (21.6%)		1730 (19.3%)	3645 (20.4%)	
25 - <30	740 (25.2%)	1591 (27.0%)		2377 (26.6%)	4609 (25.8%)	
30 - <35	590 (20.1%)	1084 (18.4%)		1655 (18.5%)	3503 (19.6%)	
35 - <40	295 (10.0%)	607 (10.3%)		837 (9.4%)	1806 (10.1%)	
40 - <45	117 (4.0%)	247 (4.2%)		393 (4.4%)	795 (4.4%)	
≥45	66 (2.2%)	147 (2.5%)		245 (2.7%)	539 (3.0%)	
Unknown	569 (19.3%)	864 (14.7%)		1605 (17.9%)	2773 (15.5%)	
Smoking[c], n (%)			<0.01 / 0.12			<0.01 / 0.07
No	1981 (67.3%)	4103 (69.7%)		6375 (71.3%)	12709 (71.0%)	
Yes	484 (16.5%)	1068 (18.2%)		1268 (14.2%)	2883 (16.1%)	
Unknown	477 (16.2%)	713 (12.1%)		1304 (14.6%)	2302 (12.9%)	
Charlson comorbidity score[b], n (%)			0.01 / 0.07			<0.01 / 0.13
0	2443 (83.0%)	4760 (80.9%)		7721 (86.3%)	14725 (82.3%)	
1	316 (10.7%)	652 (11.1%)		866 (9.7%)	2001 (11.2%)	
≥2	183 (6.2%)	472 (8.0%)		360 (4.0%)	1168 (6.5%)	

Frailty index[b], n (%)			<0.01 / 0.17			<0.01 / 0.13
Quartile 1	731 (24.8%)	1474 (25.1%)		2163 (24.2%)	4258 (23.8%)	
Quartile 2	856 (29.1%)	1350 (22.9%)		2571 (28.7%)	4424 (24.7%)	
Quartile 3	728 (24.7%)	1481 (25.2%)		2272 (25.4%)	4442 (24.8%)	
Quartile 4 (most frail)	627 (21.3%)	1579 (26.8%)		1941 (21.7%)	4770 (26.7%)	
Chronic diseases[b], n (%)						
Kidney disease	41 (1.4%)	117 (2.0%)	0.05 / 0.05	62 (0.7%)	289 (1.6%)	<0.01 / 0.09
Heart disease	34 (1.2%)	108 (1.8%)	0.02 / 0.06	59 (0.7%)	232 (1.3%)	<0.01 / 0.06
Lung disease	193 (6.6%)	461 (7.8%)	0.03 / 0.05	531 (5.9%)	1393 (7.8%)	<0.01 / 0.07
Liver disease	68 (2.3%)	162 (2.8%)	0.22 / 0.03	175 (2.0%)	483 (2.7%)	<0.01 / 0.05
Diabetes	170 (5.8%)	386 (6.6%)	0.15 / 0.03	398 (4.4%)	1064 (5.9%)	<0.01 / 0.07
Immunocompromised, n (%)	32 (1.1%)	173 (2.9%)	<0.01 / 0.13	131 (1.5%)	488 (2.7%)	<0.01 / 0.09
HIV/AIDS	2	4		12	30	
Leukemia/lymphoma, congenital and other immunodeficiencies, asplenia/hyposplenia	12	62		44	150	
Hematopoietic stem cell transplantation/ organ transplant	2	6		7	29	
Immunosuppressant medications	18	120		83	342	
Autoimmune conditions[b], n (%)	55 (1.9%)	160 (2.7%)	0.01 / 0.06	146 (1.6%)	373 (2.1%)	0.01 / 0.03
Rheumatoid arthritis	13	54		49	165	
Inflammatory bowel disease	15	50		42	92	
Psoriasis and psoriatic arthritis	23	53		44	94	
Multiple sclerosis	4	8		12	24	
Systemic lupus erythematosus	3	12		19	55	
Pregnant at specimen collection date, n (%)	57 (1.9%)	203 (3.5%)	<0.01 / 0.09	195 (2.2%)	774 (4.3%)	<0.01 / 0.12
1st trimester	16	28		33	80	
2nd trimester	19	48		69	174	
3rd trimester	22	127		93	520	
History of COVID-19[d], n (%)	79 (2.7%)	1354 (23.0%)	<0.01 / 0.64	1622 (18.1%)	4473 (25.0%)	<0.01 / 0.17
History of SARS-CoV-2 molecular test[d], n (%)	1886 (64.1%)	4684 (79.6%)	<0.01 / 0.35	6563 (73.4%)	13347 (74.6%)	0.03 / 0.03
Number of outpatient and virtual visits[a], n (%)			<0.01 / 0.29			<0.01 / 0.19
0	460 (15.6%)	586 (10.0%)		1196 (13.4%)	1920 (10.7%)	
1-4	1123 (38.2%)	1823 (31.0%)		3460 (38.7%)	5990 (33.5%)	
5-10	711 (24.2%)	1593 (27.1%)		2275 (25.4%)	4596 (25.7%)	
≥11	648 (22.0%)	1882 (32.0%)		2016 (22.5%)	5388 (30.1%)	
Number of Emergency Department visits[b], n (%)			<0.01 / 0.18			<0.01 / 0.14
0	2513 (85.4%)	4642 (78.9%)		7449 (83.3%)	14106 (78.8%)	
1	308 (10.5%)	830 (14.1%)		1111 (12.4%)	2506 (14.0%)	

≥2	121 (4.1%)	412 (7.0%)		387 (4.3%)	1282 (7.2%)	
Number of hospitalizations[b], n (%)			0.02 / 0.07			<0.01 / 0.11
0	2797 (95.1%)	5512 (93.7%)		8561 (95.7%)	16710 (93.4%)	
1	118 (4.0%)	285 (4.8%)		340 (3.8%)	948 (5.3%)	
≥2	27 (0.9%)	87 (1.5%)		46 (0.5%)	236 (1.3%)	
Preventive care[b], n(%)	1355 (46.1%)	2965 (50.4%)	<0.01 / 0.09	3971 (44.4%)	8578 (47.9%)	<0.01 / 0.07
Medicaid, n (%)	303 (10.3%)	684 (11.6%)	0.06 / 0.04	1077 (12.0%)	2495 (13.9%)	<0.01 / 0.06
Neighborhood median household income, n(%)			0.04 / 0.07			<0.01 / 0.06
< $40,000	127 (4.3%)	273 (4.6%)		387 (4.3%)	948 (5.3%)	
$40,000-$59,999	490 (16.7%)	1132 (19.2%)		1789 (20.0%)	3820 (21.3%)	
$60,000-$79,999	799 (27.2%)	1515 (25.7%)		2447 (27.3%)	4724 (26.4%)	
$80,000+	1524 (51.8%)	2959 (50.3%)		4307 (48.1%)	8373 (46.8%)	
Unknown	2 (0.1%)	5 (0.1%)		17 (0.2%)	29 (0.2%)	
KPSC physician/employee, n (%)	75 (2.5%)	533 (9.1%)	<0.01 / 0.28	275 (3.1%)	466 (2.6%)	0.03 / 0.03
Specimen type, n (%)			<0.01 / 0.55			<0.01 / 0.21
Nasopharyngeal/oropharyngeal swab	2541 (86.4%)	3724 (63.3%)		7599 (84.9%)	13730 (76.7%)	
Saliva	401 (13.6%)	2160 (36.7%)		1348 (15.1%)	4164 (23.3%)	

[a] Statistical tests were two-sided and no adjustments were made for multiple comparison.

[b] Defined in the one year prior to specimen collection date

[c] Defined in the two years prior to specimen collection date

[d] Defined based on all available medical records from March 1, 2020 to specimen collection date

Medical center area not shown. There were differences in the distribution of the vaccinated and unvaccinated individuals across the 19 medical center areas.

N/A = not applicable

Supplementary Table 2. Full model for 1-dose VE against delta variant infection

Odds Ratio Estimates			
Effect	Point Estimate	95% Wald Confidence Interval	
Vaccination status, Vaccinated vs Unvaccinated	0.433	0.316	0.593
History of SARS-CoV-2 molecular test, No vs Yes	1.347	1.201	1.512
Preventive Care, No vs Yes	1.12	0.995	1.261
Number of outpatient and virtual visits, 0 vs >=11	1.581	1.236	2.022
Number of outpatient and virtual visits, 1-4 vs >=11	1.443	1.236	1.686
Number of outpatient and virtual visits, 5-10 vs >=11	1.151	0.991	1.336
Obese, No vs Yes	0.857	0.764	0.961
Obese, Unknown vs Yes	0.914	0.768	1.088
Charlson comorbidity score, 0 vs >=2	0.969	0.766	1.226
Charlson comorbidity score, 1 vs >=2	1.117	0.866	1.443
Frailty, Quartile 1 vs Quartile 4	1.227	1.04	1.448
Frailty, Quartile 2 vs Quartile 4	1.212	0.999	1.469
Frailty, Quartile 3 vs Quartile 4	1.2	1.023	1.407
Specimen type, Nasopharyngeal/oropharyngeal swab vs Saliva	3.657	3.206	4.172
Immunocompromised status, No vs Yes	2.479	1.622	3.789
History of COVID-19 diagnosis, No vs Yes	10.064	7.838	12.922

Supplementary Table 3. Full model for 1-dose VE against omicron variant infection

Odds Ratio Estimates			
**Effect**	**Point Estimate**	**95% Wald Confidence Interval**	
Vaccination status, Vaccinated vs Unvaccinated	0.796	0.7	0.905
History of SARS-CoV-2 molecular test, No vs Yes	0.849	0.797	0.904
Preventive Care, No vs Yes	1.067	1.004	1.134
Number of outpatient and virtual visits, 0 vs >=11	1.546	1.362	1.754
Number of outpatient and virtual visits, 1-4 vs >=11	1.438	1.328	1.558
Number of outpatient and virtual visits, 5-10 vs >=11	1.265	1.172	1.366
Obese, No vs Yes	1.014	0.956	1.075
Obese, Unknown vs Yes	0.984	0.9	1.076
Charlson comorbidity score, 0 vs >=2	1.394	1.212	1.604
Charlson comorbidity score, 1 vs >=2	1.295	1.113	1.507
Frailty, Quartile 1 vs Quartile 4	1.078	0.991	1.173
Frailty, Quartile 2 vs Quartile 4	1.105	1.004	1.215
Frailty, Quartile 3 vs Quartile 4	1.109	1.024	1.2
Specimen type, Nasopharyngeal/oropharyngeal swab vs Saliva	1.781	1.662	1.909
Immunocompromised status, No vs Yes	1.512	1.235	1.851
History of COVID-19 diagnosis, No vs Yes	1.53	1.43	1.637

Supplementary Table 4. Full model for 2-dose VE against delta variant infection

Odds Ratio Estimates			
Effect	Point Estimate	95% Wald Confidence Interval	
Vaccination status, Vaccinated vs Unvaccinated	0.364	0.331	0.401
History of SARS-CoV-2 molecular test, No vs Yes	1.222	1.108	1.347
Preventive Care, No vs Yes	1.064	0.96	1.178
Number of outpatient and virtual visits, 0 vs >=11	1.541	1.243	1.909
Number of outpatient and virtual visits, 1-4 vs >=11	1.425	1.248	1.628
Number of outpatient and virtual visits, 5-10 vs >=11	1.188	1.053	1.341
Obese, No vs Yes	0.882	0.801	0.971
Obese, Unknown vs Yes	0.913	0.784	1.063
Charlson comorbidity score, 0 vs >=2	1.154	0.958	1.389
Charlson comorbidity score, 1 vs >=2	1.309	1.069	1.603
Frailty, Quartile 1 vs Quartile 4	1.209	1.049	1.394
Frailty, Quartile 2 vs Quartile 4	1.205	1.033	1.405
Frailty, Quartile 3 vs Quartile 4	1.173	1.025	1.341
Specimen type, Nasopharyngeal/oropharyngeal swab vs Saliva	3.455	3.063	3.897
Immunocompromised status, No vs Yes	1.635	1.203	2.224
History of COVID-19 diagnosis, No vs Yes	10.112	8.142	12.558

Supplementary Table 5. Full model for 2-dose VE against delta variant infection by time since vaccination

Odds Ratio Estimates			
Effect	Point Estimate	95% Wald Confidence Interval	
Vaccination status, Vaccinated 14-90 days vs Unvaccinated	0.198	0.123	0.318
Vaccination status, Vaccinated 91-180 days vs Unvaccinated	0.311	0.242	0.399
Vaccination status, Vaccinated 181-270 days vs Unvaccinated	0.363	0.328	0.402
Vaccination status, Vaccinated 271-365 days vs Unvaccinated	0.387	0.333	0.45
History of SARS-CoV-2 molecular test, No vs Yes	1.23	1.12	1.352
Preventive Care, No vs Yes	1.07	0.971	1.179
Number of outpatient and virtual visits, 0 vs >=11	1.459	1.189	1.79
Number of outpatient and virtual visits, 1-4 vs >=11	1.405	1.239	1.594
Number of outpatient and virtual visits, 5-10 vs >=11	1.177	1.049	1.32
Obese, No vs Yes	0.906	0.827	0.993
Obese, Unknown vs Yes	0.952	0.824	1.1
Charlson comorbidity score, 0 vs >=2	1.126	0.944	1.344
Charlson comorbidity score, 1 vs >=2	1.235	1.02	1.497
Frailty, Quartile 1 vs Quartile 4	1.177	1.027	1.348
Frailty, Quartile 2 vs Quartile 4	1.15	0.993	1.332
Frailty, Quartile 3 vs Quartile 4	1.125	0.99	1.279
Specimen type, Nasopharyngeal/oropharyngeal swab vs Saliva	3.575	3.189	4.008
Immunocompromised status, No vs Yes	1.731	1.293	2.317
History of COVID-19 diagnosis, No vs Yes	9.929	8.06	12.231
Age group, 18-44 years old vs 75 and older	0.735	0.533	1.013
Age group, 45-64 years old vs 75 and older	0.844	0.615	1.159
Age group, 65-74 years old vs 75 and older	0.865	0.616	1.214
Sex, Female vs Male	1.185	1.087	1.292
Race/ethnicity, Hispanic vs Other/Unknown	1.157	0.986	1.358
Race/ethnicity, Non-Hispanic Asian vs Other/Unknown	1.336	1.047	1.706
Race/ethnicity, Non-Hispanic Black vs Other/Unknown	1.039	0.829	1.303
Race/ethnicity, Non-Hispanic White vs Other/Unknown	1.067	0.907	1.254

Supplementary Table 6. Full model for 2-dose VE against omicron variant infection

Odds Ratio Estimates			
Effect	Point Estimate	95% Wald Confidence Interval	
Vaccination status, Vaccinated vs Unvaccinated	0.861	0.829	0.895
History of SARS-CoV-2 molecular test, No vs Yes	0.943	0.905	0.984
Preventive Care, No vs Yes	1.12	1.075	1.168
Number of outpatient and virtual visits, 0 vs >=11	1.413	1.287	1.552
Number of outpatient and virtual visits, 1-4 vs >=11	1.379	1.305	1.457
Number of outpatient and virtual visits, 5-10 vs >=11	1.267	1.206	1.33
Obese, No vs Yes	0.942	0.905	0.98
Obese, Unknown vs Yes	0.985	0.925	1.05
Charlson comorbidity score, 0 vs >=2	1.314	1.208	1.43
Charlson comorbidity score, 1 vs >=2	1.274	1.162	1.396
Frailty, Quartile 1 vs Quartile 4	1.091	1.031	1.155
Frailty, Quartile 2 vs Quartile 4	1.099	1.031	1.171
Frailty, Quartile 3 vs Quartile 4	1.101	1.044	1.162
Specimen type, Nasopharyngeal/oropharyngeal swab vs Saliva	1.892	1.796	1.993
Immunocompromised status, No vs Yes	1.34	1.178	1.524
History of COVID-19 diagnosis, No vs Yes	1.639	1.558	1.724

Supplementary Table 7. Full model for 2-dose VE against omicron variant infection by time since vaccination

Odds Ratio Estimates			
Effect	Point Estimate	95% Wald Confidence Interval	
Vaccination status, Vaccinated 14-90 days vs Unvaccinated	0.56	0.484	0.649
Vaccination status, Vaccinated 91-180 days vs Unvaccinated	0.765	0.7	0.836
Vaccination status, Vaccinated 181-270 days vs Unvaccinated	0.862	0.827	0.898
Vaccination status, Vaccinated 271-365 days vs Unvaccinated	0.941	0.89	0.996
History of SARS-CoV-2 molecular test, No vs Yes	0.939	0.901	0.979
Preventive Care, No vs Yes	1.129	1.083	1.177
Number of outpatient and virtual visits, 0 vs >=11	1.396	1.271	1.532
Number of outpatient and virtual visits, 1-4 vs >=11	1.368	1.296	1.445
Number of outpatient and virtual visits, 5-10 vs >=11	1.26	1.2	1.322
Obese, No vs Yes	0.94	0.904	0.978
Obese, Unknown vs Yes	0.988	0.928	1.051
Charlson comorbidity score, 0 vs >=2	1.312	1.206	1.428
Charlson comorbidity score, 1 vs >=2	1.268	1.157	1.389
Frailty, Quartile 1 vs Quartile 4	1.098	1.037	1.162
Frailty, Quartile 2 vs Quartile 4	1.102	1.035	1.174
Frailty, Quartile 3 vs Quartile 4	1.105	1.047	1.166
Specimen type, Nasopharyngeal/oropharyngeal swab vs Saliva	1.897	1.801	1.998
Immunocompromised status, No vs Yes	1.344	1.182	1.528
History of COVID-19 diagnosis, No vs Yes	1.638	1.557	1.723
Age group, 18-44 years old vs 75 and older	0.729	0.609	0.872
Age group, 45-64 years old vs 75 and older	0.818	0.684	0.978
Age group, 65-74 years old vs 75 and older	0.902	0.742	1.097
Sex, Female vs Male	1.106	1.066	1.148
Race/ethnicity, Hispanic vs Other/Unknown	1.067	0.999	1.14
Race/ethnicity, Non-Hispanic Asian vs Other/Unknown	1.049	0.961	1.144
Race/ethnicity, Non-Hispanic Black vs Other/Unknown	1.092	1.003	1.188
Race/ethnicity, Non-Hispanic White vs Other/Unknown	1.05	0.979	1.126

Supplementary Table 8. Full model for 3-dose VE against delta variant infection

Odds Ratio Estimates			
Effect	Point Estimate	95% Wald Confidence Interval	
Vaccination status, Vaccinated vs Unvaccinated	0.055	0.043	0.071
History of SARS-CoV-2 molecular test, No vs Yes	1.391	1.226	1.578
Preventive Care, No vs Yes	1.176	1.031	1.342
Number of outpatient and virtual visits, 0 vs >=11	1.434	1.105	1.861
Number of outpatient and virtual visits, 1-4 vs >=11	1.407	1.187	1.667
Number of outpatient and virtual visits, 5-10 vs >=11	1.138	0.97	1.336
Obese, No vs Yes	0.881	0.78	0.996
Obese, Unknown vs Yes	0.92	0.762	1.111
Charlson comorbidity score, 0 vs >=2	0.951	0.737	1.227
Charlson comorbidity score, 1 vs >=2	1.216	0.921	1.606
Frailty, Quartile 1 vs Quartile 4	1.145	0.953	1.374
Frailty, Quartile 2 vs Quartile 4	1.042	0.852	1.275
Frailty, Quartile 3 vs Quartile 4	1.051	0.884	1.249
Specimen type, Nasopharyngeal/oropharyngeal swab vs Saliva	3.673	3.191	4.229
Immunocompromised status, No vs Yes	1.935	1.298	2.884
History of COVID-19 diagnosis, No vs Yes	7.863	6.188	9.992

Supplementary Table 9. Full model for 3-dose VE against delta variant infection by time since vaccination

Effect	Point Estimate	95% Wald Confidence Interval	
Vaccination status, Vaccinated 14-60 days vs Unvaccinated	0.063	0.051	0.078
Vaccination status, Vaccinated 61-365 days vs Unvaccinated	0.14	0.089	0.219
History of SARS-CoV-2 molecular test, No vs Yes	1.4	1.246	1.572
Preventive Care, No vs Yes	1.154	1.022	1.303
Number of outpatient and virtual visits, 0 vs >=11	1.453	1.14	1.851
Number of outpatient and virtual visits, 1-4 vs >=11	1.414	1.209	1.653
Number of outpatient and virtual visits, 5-10 vs >=11	1.175	1.013	1.362
Obese, No vs Yes	0.869	0.777	0.973
Obese, Unknown vs Yes	0.947	0.797	1.125
Charlson comorbidity score, 0 vs >=2	0.972	0.77	1.226
Charlson comorbidity score, 1 vs >=2	1.214	0.943	1.561
Frailty, Quartile 1 vs Quartile 4	1.151	0.973	1.362
Frailty, Quartile 2 vs Quartile 4	1.07	0.889	1.289
Frailty, Quartile 3 vs Quartile 4	1.06	0.905	1.242
Specimen type, Nasopharyngeal/oropharyngeal swab vs Saliva	3.804	3.35	4.319
Immunocompromised status, No vs Yes	1.982	1.376	2.855
History of COVID-19 diagnosis, No vs Yes	8.137	6.498	10.191
Age group, 18-44 years old vs 75 and older	0.317	0.203	0.494
Age group, 45-64 years old vs 75 and older	0.432	0.278	0.669
Age group, 65-74 years old vs 75 and older	0.763	0.479	1.216
Sex, Female vs Male	1.26	1.132	1.402
Race/ethnicity, Hispanic vs Other/Unknown	0.971	0.801	1.178
Race/ethnicity, Non-Hispanic Asian vs Other/Unknown	1.964	1.414	2.726
Race/ethnicity, Non-Hispanic Black vs Other/Unknown	0.906	0.694	1.184
Race/ethnicity, Non-Hispanic White vs Other/Unknown	1.058	0.87	1.287

Odds Ratio Estimates

Supplementary Table 10. Full model for 3-dose VE against omicron variant infection

Odds Ratio Estimates			
Effect	Point Estimate	95% Wald Confidence Interval	
Vaccination status, Vaccinated vs Unvaccinated	0.3	0.281	0.32
History of SARS-CoV-2 molecular test, No vs Yes	0.917	0.865	0.972
Preventive Care, No vs Yes	1.073	1.011	1.14
Number of outpatient and virtual visits, 0 vs >=11	1.566	1.384	1.772
Number of outpatient and virtual visits, 1-4 vs >=11	1.377	1.278	1.484
Number of outpatient and virtual visits, 5-10 vs >=11	1.208	1.13	1.291
Obese, No vs Yes	1.019	0.965	1.076
Obese, Unknown vs Yes	0.984	0.901	1.074
Charlson comorbidity score, 0 vs >=2	1.092	0.981	1.216
Charlson comorbidity score, 1 vs >=2	1.003	0.894	1.125
Frailty, Quartile 1 vs Quartile 4	1.053	0.973	1.141
Frailty, Quartile 2 vs Quartile 4	1.053	0.966	1.148
Frailty, Quartile 3 vs Quartile 4	1.091	1.012	1.175
Specimen type, Nasopharyngeal/oropharyngeal swab vs Saliva	1.661	1.558	1.771
Immunocompromised status, No vs Yes	1.105	0.95	1.285
History of COVID-19 diagnosis, No vs Yes	1.469	1.373	1.572

Supplementary Table 11. Full model for 3-dose VE against omicron variant infection by time since vaccination

Odds Ratio Estimates			
**Effect**	**Point Estimate**	**95% Wald Confidence Interval**	
Vaccination status, Vaccinated 14-60 days vs Unvaccinated	0.284	0.266	0.303
Vaccination status, Vaccinated 61-365 days vs Unvaccinated	0.526	0.465	0.595
History of SARS-CoV-2 molecular test, No vs Yes	0.916	0.865	0.97
Preventive Care, No vs Yes	1.077	1.015	1.143
Number of outpatient and virtual visits, 0 vs >=11	1.57	1.392	1.772
Number of outpatient and virtual visits, 1-4 vs >=11	1.387	1.289	1.492
Number of outpatient and virtual visits, 5-10 vs >=11	1.227	1.149	1.31
Obese, No vs Yes	1.013	0.961	1.068
Obese, Unknown vs Yes	0.975	0.894	1.062
Charlson comorbidity score, 0 vs >=2	1.119	1.007	1.244
Charlson comorbidity score, 1 vs >=2	1.021	0.911	1.143
Frailty, Quartile 1 vs Quartile 4	1.063	0.983	1.149
Frailty, Quartile 2 vs Quartile 4	1.061	0.974	1.155
Frailty, Quartile 3 vs Quartile 4	1.089	1.013	1.172
Specimen type, Nasopharyngeal/oropharyngeal swab vs Saliva	1.685	1.582	1.794
Immunocompromised status, No vs Yes	1.217	1.048	1.413
History of COVID-19 diagnosis, No vs Yes	1.477	1.382	1.579
Age group, 18-44 years old vs 75 and older	0.42	0.352	0.502
Age group, 45-64 years old vs 75 and older	0.599	0.503	0.714
Age group, 65-74 years old vs 75 and older	0.887	0.736	1.069
Sex, Female vs Male	1.135	1.079	1.193
Race/ethnicity, Hispanic vs Other/Unknown	0.977	0.891	1.071
Race/ethnicity, Non-Hispanic Asian vs Other/Unknown	1.443	1.274	1.635
Race/ethnicity, Non-Hispanic Black vs Other/Unknown	0.939	0.839	1.052
Race/ethnicity, Non-Hispanic White vs Other/Unknown	1.015	0.921	1.117

Supplementary Table 12. Full model for 3-dose VE against delta variant infection by time since vaccination (excluding immunocompromised population)

Odds Ratio Estimates			
Effect	Point Estimate	95% Wald Confidence Interval	
Vaccination status, Vaccinated 14-60 days vs Unvaccinated	0.058	0.047	0.073
Vaccination status, Vaccinated 61-365 days vs Unvaccinated	0.119	0.071	0.198
History of SARS-CoV-2 molecular test, No vs Yes	1.388	1.234	1.561
Preventive Care, No vs Yes	1.144	1.012	1.293
Number of outpatient and virtual visits, 0 vs >=11	1.467	1.148	1.876
Number of outpatient and virtual visits, 1-4 vs >=11	1.429	1.22	1.674
Number of outpatient and virtual visits, 5-10 vs >=11	1.193	1.026	1.386
Obese, No vs Yes	0.861	0.768	0.965
Obese, Unknown vs Yes	0.933	0.784	1.109
Charlson comorbidity score, 0 vs >=2	0.945	0.742	1.204
Charlson comorbidity score, 1 vs >=2	1.159	0.891	1.507
Frailty, Quartile 1 vs Quartile 4	1.157	0.977	1.37
Frailty, Quartile 2 vs Quartile 4	1.093	0.904	1.32
Frailty, Quartile 3 vs Quartile 4	1.096	0.933	1.287
Specimen type, Nasopharyngeal/oropharyngeal swab vs Saliva	3.79	3.335	4.307
History of COVID-19 diagnosis, No vs Yes	8.248	6.569	10.356
Age group, 18-44 years old vs 75 and older	0.308	0.195	0.489
Age group, 45-64 years old vs 75 and older	0.419	0.266	0.661
Age group, 65-74 years old vs 75 and older	0.786	0.485	1.275
Sex, Female vs Male	1.264	1.134	1.408
Race/ethnicity, Hispanic vs Other/Unknown	0.97	0.799	1.179
Race/ethnicity, Non-Hispanic Asian vs Other/Unknown	1.96	1.404	2.735
Race/ethnicity, Non-Hispanic Black vs Other/Unknown	0.881	0.673	1.154
Race/ethnicity, Non-Hispanic White vs Other/Unknown	1.07	0.879	1.304

Supplementary Table 13. Full model for 3-dose VE against omicron variant infection by time since vaccination (excluding immunocompromised population)

Odds Ratio Estimates			
**Effect**	**Point Estimate**	**95% Wald Confidence Interval**	
Vaccination status, Vaccinated 14-60 days vs Unvaccinated	0.279	0.261	0.298
Vaccination status, Vaccinated 61-365 days vs Unvaccinated	0.488	0.427	0.558
History of SARS-CoV-2 molecular test, No vs Yes	0.91	0.859	0.964
Preventive Care, No vs Yes	1.067	1.004	1.133
Number of outpatient and virtual visits, 0 vs >=11	1.566	1.385	1.769
Number of outpatient and virtual visits, 1-4 vs >=11	1.381	1.282	1.487
Number of outpatient and virtual visits, 5-10 vs >=11	1.217	1.138	1.301
Obese, No vs Yes	1.015	0.962	1.071
Obese, Unknown vs Yes	0.978	0.896	1.066
Charlson comorbidity score, 0 vs >=2	1.136	1.016	1.269
Charlson comorbidity score, 1 vs >=2	1.042	0.925	1.174
Frailty, Quartile 1 vs Quartile 4	1.065	0.985	1.153
Frailty, Quartile 2 vs Quartile 4	1.066	0.978	1.163
Frailty, Quartile 3 vs Quartile 4	1.089	1.011	1.172
Specimen type, Nasopharyngeal/oropharyngeal swab vs Saliva	1.704	1.599	1.817
History of COVID-19 diagnosis, No vs Yes	1.478	1.382	1.581
Age group, 18-44 years old vs 75 and older	0.399	0.332	0.481
Age group, 45-64 years old vs 75 and older	0.568	0.473	0.683
Age group, 65-74 years old vs 75 and older	0.854	0.704	1.037
Sex, Female vs Male	1.139	1.083	1.199
Race/ethnicity, Hispanic vs Other/Unknown	0.973	0.887	1.069
Race/ethnicity, Non-Hispanic Asian vs Other/Unknown	1.461	1.287	1.658
Race/ethnicity, Non-Hispanic Black vs Other/Unknown	0.937	0.836	1.051
Race/ethnicity, Non-Hispanic White vs Other/Unknown	1.015	0.92	1.118

Supplementary Table 14. Full model for 1-dose VE against delta variant hospitalization

Odds Ratio Estimates			
Effect	Point Estimate	95% Wald Confidence Interval	
Vaccination status, Vaccinated vs Unvaccinated	0.288	0.026	3.194
History of SARS-CoV-2 molecular test, No vs Yes	1.59	0.795	3.182
Preventive Care, No vs Yes	2.393	1.041	5.502
Obese, No vs Yes	0.166	0.066	0.413
Obese, Unknown vs Yes	0.268	0.082	0.882
Charlson comorbidity score, 0 vs >=2	0.592	0.237	1.481
Charlson comorbidity score, 1 vs >=2	0.902	0.282	2.88
History of COVID-19 diagnosis, No vs Yes	10.386	2.246	48.021

Supplementary Table 15. Full model for 2-dose VE against delta variant hospitalization

Odds Ratio Estimates			
Effect	Point Estimate	95% Wald Confidence Interval	
Vaccination status, Vaccinated vs Unvaccinated	0.01	0.001	0.067
History of SARS-CoV-2 molecular test, No vs Yes	0.734	0.267	2.02
Preventive Care, No vs Yes	4.08	1.353	12.307
Obese, No vs Yes	0.092	0.024	0.349
Obese, Unknown vs Yes	0.546	0.132	2.265
Charlson comorbidity score, 0 vs >=2	0.219	0.039	1.224
Charlson comorbidity score, 1 vs >=2	1.259	0.197	8.031
History of COVID-19 diagnosis, No vs Yes	18.718	2.917	120.099

Supplementary Table 16. Full model for 2-dose VE against omicron variant hospitalization

Odds Ratio Estimates

Effect	Point Estimate	95% Wald Confidence Interval	
Vaccination status, Vaccinated vs Unvaccinated	0.155	0.031	0.77
History of SARS-CoV-2 molecular test, No vs Yes	4.677	0.635	34.442
Preventive Care, No vs Yes	0.717	0.146	3.515
Charlson comorbidity score, 0 vs >=2	0.902	0.083	9.842
Charlson comorbidity score, 1 vs >=2	3.359	0.237	47.543
Immunocompromised status, No vs Yes	0.175	0.006	5.289
History of COVID-19 diagnosis, No vs Yes	2.364	0.172	32.54

Supplementary Table 17. Full model for 3-dose VE against delta variant hospitalization

Odds Ratio Estimates

Effect	Point Estimate	95% Wald Confidence Interval	
Vaccination status, Vaccinated vs Unvaccinated	0.003	<0.001	0.035
History of SARS-CoV-2 molecular test, No vs Yes	1.738	0.79	3.825
Preventive Care, No vs Yes	1.715	0.759	3.875
Obese, No vs Yes	0.322	0.136	0.762
Obese, Unknown vs Yes	0.418	0.14	1.249
Charlson comorbidity score, 0 vs >=2	0.313	0.105	0.931
Charlson comorbidity score, 1 vs >=2	0.479	0.131	1.753
History of COVID-19 diagnosis, No vs Yes	8.736	1.711	44.601
Age group, 18-44 years old vs 75 and older	0.202	0.031	1.312
Age group, 45-64 years old vs 75 and older	0.23	0.039	1.363
Age group, 65-74 years old vs 75 and older	1.484	0.177	12.452
Sex, Female vs Male	1.157	0.53	2.524
Race/ethnicity, Hispanic vs Other/Unknown	0.817	0.202	3.304
Race/ethnicity, Non-Hispanic Asian vs Other/Unknown	1.146	0.138	9.498
Race/ethnicity, Non-Hispanic Black vs Other/Unknown	1.428	0.254	8.024
Race/ethnicity, Non-Hispanic White vs Other/Unknown	0.858	0.202	3.638

Supplementary Table 18. Full model for 3-dose VE against omicron variant hospitalization

Odds Ratio Estimates			
Effect	Point Estimate	95% Wald Confidence Interval	
Vaccination status, Vaccinated vs Unvaccinated	0.008	<0.001	0.237
History of SARS-CoV-2 molecular test, No vs Yes	0.473	0.059	3.765
Preventive Care, No vs Yes	1.008	0.075	13.531
Charlson comorbidity score, 0 vs >=2	0.033	0.002	0.725
Charlson comorbidity score, 1 vs >=2	0.765	0.02	29.284
Immunocompromised status, No vs Yes	0.261	0.007	9.422
Age group, 18-44 years old vs 75 and older	0.862	0.052	14.438
Age group, 45-64 years old vs 75 and older	2.605	0.099	68.699
Age group, 65-74 years old vs 75 and older	1.62	0.078	33.492
Sex, Female vs Male	0.216	0.02	2.356
Race/ethnicity, Hispanic vs Non-Hispanic White	3.084	0.2	47.486
Race/ethnicity, Non-Hispanic Asian vs Non-Hispanic White	5.291	0.124	224.949
Race/ethnicity, Non-Hispanic Black vs Non-Hispanic White	0.435	0.024	7.917

Supplementary Table 19. Full model for 3-dose VE against delta variant infection (subgroup of age<65)

Odds Ratio Estimates			
Effect	Point Estimate	95% Wald Confidence Interval	
Vaccination status, Vaccinated vs Unvaccinated	0.057	0.043	0.075
History of SARS-CoV-2 molecular test, No vs Yes	1.416	1.243	1.614
Preventive Care, No vs Yes	1.18	1.032	1.349
Number of outpatient and virtual visits, 0 vs >=11	1.42	1.084	1.86
Number of outpatient and virtual visits, 1-4 vs >=11	1.412	1.186	1.682
Number of outpatient and virtual visits, 5-10 vs >=11	1.158	0.981	1.368
Obese, No vs Yes	0.895	0.788	1.016
Obese, Unknown vs Yes	0.927	0.765	1.123
Charlson comorbidity score, 0 vs >=2	0.939	0.707	1.246
Charlson comorbidity score, 1 vs >=2	1.186	0.866	1.623
Frailty, Quartile 1 vs Quartile 4	1.176	0.981	1.41
Frailty, Quartile 2 vs Quartile 4	1.1	0.893	1.356
Frailty, Quartile 3 vs Quartile 4	1.096	0.921	1.306
Specimen type, Nasopharyngeal/oropharyngeal swab vs Saliva	3.833	3.315	4.432
Immunocompromised status, No vs Yes	1.754	1.14	2.699
History of COVID-19 diagnosis, No vs Yes	7.724	6.063	9.84

Supplementary Table 20. Full model for 3-dose VE against delta variant infection (subgroup of age≥65)

Odds Ratio Estimates			
Effect	Point Estimate	95% Wald Confidence Interval	
Vaccination status, Vaccinated vs Unvaccinated	0.04	0.021	0.077
History of SARS-CoV-2 molecular test, No vs Yes	1.127	0.674	1.884
Preventive Care, No vs Yes	1.119	0.522	2.399
Number of outpatient and virtual visits, 0 vs >=11	0.945	0.156	5.734
Number of outpatient and virtual visits, 1-4 vs >=11	1.002	0.441	2.276
Number of outpatient and virtual visits, 5-10 vs >=11	0.682	0.365	1.276
Obese, No vs Yes	0.633	0.378	1.062
Obese, Unknown vs Yes	0.671	0.175	2.579
Charlson comorbidity score, 0 vs >=2	0.814	0.401	1.653
Charlson comorbidity score, 1 vs >=2	1.259	0.639	2.482
Frailty, Quartile 1 vs Quartile 4	2.186	0.942	5.076
Frailty, Quartile 2 vs Quartile 4	2.095	0.962	4.564
Frailty, Quartile 3 vs Quartile 4	2.14	1.011	4.527
Specimen type, Nasopharyngeal/oropharyngeal swab vs Saliva	1.503	0.771	2.93
Immunocompromised status, No vs Yes	4.096	1.381	12.154
History of COVID-19 diagnosis, No vs Yes	10.522	2.028	54.586

Supplementary Table 21. Full model for 3-dose VE against delta variant infection (subgroup of female)

Odds Ratio Estimates			
Effect	Point Estimate	95% Wald Confidence Interval	
Vaccination status, Vaccinated vs Unvaccinated	0.056	0.04	0.078
History of SARS-CoV-2 molecular test, No vs Yes	1.411	1.172	1.7
Preventive Care, No vs Yes	1.123	0.94	1.343
Number of outpatient and virtual visits, 0 vs >=11	1.863	1.27	2.732
Number of outpatient and virtual visits, 1-4 vs >=11	1.464	1.173	1.828
Number of outpatient and virtual visits, 5-10 vs >=11	1.33	1.086	1.629
Obese, No vs Yes	0.895	0.76	1.052
Obese, Unknown vs Yes	0.765	0.568	1.031
Charlson comorbidity score, 0 vs >=2	1.004	0.708	1.425
Charlson comorbidity score, 1 vs >=2	1.16	0.796	1.69
Frailty, Quartile 1 vs Quartile 4	1.075	0.837	1.381
Frailty, Quartile 2 vs Quartile 4	0.95	0.73	1.236
Frailty, Quartile 3 vs Quartile 4	1.014	0.8	1.286
Specimen type, Nasopharyngeal/oropharyngeal swab vs Saliva	3.769	3.115	4.56
Immunocompromised status, No vs Yes	1.606	0.97	2.662
History of COVID-19 diagnosis, No vs Yes	7.264	5.311	9.937

Supplementary Table 22. Full model for 3-dose VE against delta variant infection (subgroup of male)

Odds Ratio Estimates			
Effect	Point Estimate	95% Wald Confidence Interval	
Vaccination status, Vaccinated vs Unvaccinated	0.054	0.037	0.08
History of SARS-CoV-2 molecular test, No vs Yes	1.392	1.171	1.654
Preventive Care, No vs Yes	1.249	1.024	1.524
Number of outpatient and virtual visits, 0 vs >=11	1.141	0.798	1.633
Number of outpatient and virtual visits, 1-4 vs >=11	1.306	0.992	1.72
Number of outpatient and virtual visits, 5-10 vs >=11	0.914	0.699	1.195
Obese, No vs Yes	0.871	0.721	1.052
Obese, Unknown vs Yes	1.027	0.8	1.32
Charlson comorbidity score, 0 vs >=2	0.886	0.608	1.292
Charlson comorbidity score, 1 vs >=2	1.294	0.853	1.964
Frailty, Quartile 1 vs Quartile 4	1.209	0.928	1.575
Frailty, Quartile 2 vs Quartile 4	1.052	0.682	1.623
Frailty, Quartile 3 vs Quartile 4	1.114	0.871	1.423
Specimen type, Nasopharyngeal/oropharyngeal swab vs Saliva	3.54	2.868	4.368
Immunocompromised status, No vs Yes	2.598	1.356	4.979
History of COVID-19 diagnosis, No vs Yes	8.964	6.166	13.032

Supplementary Table 23. Full model for 3-dose VE against delta variant infection (subgroup of Hispanic)

Odds Ratio Estimates			
Effect	Point Estimate	95% Wald Confidence Interval	
Vaccination status, Vaccinated vs Unvaccinated	0.069	0.045	0.106
History of SARS-CoV-2 molecular test, No vs Yes	1.34	1.1	1.633
Preventive Care, No vs Yes	1.215	0.996	1.481
Number of outpatient and virtual visits, 0 vs >=11	1.496	1.009	2.217
Number of outpatient and virtual visits, 1-4 vs >=11	1.47	1.135	1.904
Number of outpatient and virtual visits, 5-10 vs >=11	1.256	0.977	1.614
Obese, No vs Yes	0.972	0.809	1.169
Obese, Unknown vs Yes	1.092	0.826	1.444
Charlson comorbidity score, 0 vs >=2	0.728	0.481	1.101
Charlson comorbidity score, 1 vs >=2	0.875	0.56	1.366
Frailty, Quartile 1 vs Quartile 4	1.096	0.832	1.443
Frailty, Quartile 2 vs Quartile 4	1.038	0.767	1.404
Frailty, Quartile 3 vs Quartile 4	1.161	0.894	1.507
Specimen type, Nasopharyngeal/oropharyngeal swab vs Saliva	4.264	3.377	5.384
Immunocompromised status, No vs Yes	2.077	1.083	3.982
History of COVID-19 diagnosis, No vs Yes	9.321	6.63	13.104

Supplementary Table 24. Full model for 3-dose VE against delta variant infection (subgroup of Non-Hispanic and others)

Odds Ratio Estimates			
**Effect**	**Point Estimate**	**95% Wald Confidence Interval**	
Vaccination status, Vaccinated vs Unvaccinated	0.049	0.036	0.068
History of SARS-CoV-2 molecular test, No vs Yes	1.452	1.231	1.712
Preventive Care, No vs Yes	1.143	0.958	1.365
Number of outpatient and virtual visits, 0 vs >=11	1.372	0.967	1.946
Number of outpatient and virtual visits, 1-4 vs >=11	1.323	1.055	1.658
Number of outpatient and virtual visits, 5-10 vs >=11	1.029	0.835	1.269
Obese, No vs Yes	0.805	0.682	0.951
Obese, Unknown vs Yes	0.792	0.611	1.026
Charlson comorbidity score, 0 vs >=2	1.117	0.806	1.549
Charlson comorbidity score, 1 vs >=2	1.515	1.058	2.169
Frailty, Quartile 1 vs Quartile 4	1.213	0.949	1.55
Frailty, Quartile 2 vs Quartile 4	1.075	0.821	1.408
Frailty, Quartile 3 vs Quartile 4	0.998	0.792	1.258
Specimen type, Nasopharyngeal/oropharyngeal swab vs Saliva	3.349	2.803	4.003
Immunocompromised status, No vs Yes	1.863	1.12	3.1
History of COVID-19 diagnosis, No vs Yes	6.581	4.69	9.236

Supplementary Table 25. Full model for 3-dose VE against delta variant infection (subgroup of immunocompromised population)

Odds Ratio Estimates

Effect	Point Estimate	95% Wald Confidence Interval	
Vaccination status, Vaccinated vs Unvaccinated	0.294	0.125	0.69
History of SARS-CoV-2 molecular test, No vs Yes	1.719	0.783	3.774
Preventive Care, No vs Yes	1.703	0.688	4.213
Obese, No vs Yes	1.364	0.619	3.003
Obese, Unknown vs Yes	3.835	0.448	32.845
Charlson comorbidity score, 0 vs >=2	0.909	0.35	2.364
Charlson comorbidity score, 1 vs >=2	2.397	0.912	6.297
Frailty, Quartile 1 vs Quartile 4	2.594	0.884	7.615
Frailty, Quartile 2 vs Quartile 4	0.856	0.276	2.658
Frailty, Quartile 3 vs Quartile 4	1.325	0.457	3.839
Specimen type, Nasopharyngeal/oropharyngeal swab vs Saliva	12.207	2.606	57.185
History of COVID-19 diagnosis, No vs Yes	7.615	1.512	38.353
Age group, 18-44 years old vs 75 and older	0.268	0.035	2.052
Age group, 45-64 years old vs 75 and older	0.355	0.05	2.514
Age group, 65-74 years old vs 75 and older	0.323	0.043	2.42
Sex, Female vs Male	1.429	0.667	3.063
Race/ethnicity, Hispanic vs Other/Unknown	1.22	0.187	7.958
Race/ethnicity, Non-Hispanic Asian vs Other/Unknown	4.203	0.408	43.27
Race/ethnicity, Non-Hispanic Black vs Other/Unknown	3.04	0.358	25.813
Race/ethnicity, Non-Hispanic White vs Other/Unknown	0.778	0.12	5.06

Supplementary Table 26. Full model for 3-dose VE against delta variant infection (subgroup of non-immunocompromised)

Odds Ratio Estimates			
Effect	Point Estimate	95% Wald Confidence Interval	
Vaccination status, Vaccinated vs Unvaccinated	0.063	0.051	0.078
History of SARS-CoV-2 molecular test, No vs Yes	1.387	1.233	1.56
Preventive Care, No vs Yes	1.144	1.011	1.293
Number of outpatient and virtual visits, 0 vs >=11	1.472	1.152	1.882
Number of outpatient and virtual visits, 1-4 vs >=11	1.43	1.221	1.676
Number of outpatient and virtual visits, 5-10 vs >=11	1.192	1.026	1.386
Obese, No vs Yes	0.861	0.768	0.966
Obese, Unknown vs Yes	0.933	0.785	1.11
Charlson comorbidity score, 0 vs >=2	0.936	0.735	1.193
Charlson comorbidity score, 1 vs >=2	1.153	0.886	1.5
Frailty, Quartile 1 vs Quartile 4	1.159	0.979	1.373
Frailty, Quartile 2 vs Quartile 4	1.092	0.904	1.319
Frailty, Quartile 3 vs Quartile 4	1.098	0.935	1.29
Specimen type, Nasopharyngeal/oropharyngeal swab vs Saliva	3.789	3.334	4.306
History of COVID-19 diagnosis, No vs Yes	8.245	6.566	10.352
Age group, 18-44 years old vs 75 and older	0.292	0.184	0.464
Age group, 45-64 years old vs 75 and older	0.397	0.251	0.626
Age group, 65-74 years old vs 75 and older	0.752	0.462	1.222
Sex, Female vs Male	1.262	1.133	1.406
Race/ethnicity, Hispanic vs Other/Unknown	0.97	0.798	1.178
Race/ethnicity, Non-Hispanic Asian vs Other/Unknown	1.969	1.411	2.747
Race/ethnicity, Non-Hispanic Black vs Other/Unknown	0.881	0.673	1.153
Race/ethnicity, Non-Hispanic White vs Other/Unknown	1.073	0.881	1.307

Supplementary Table 27. Full model for 3-dose VE against omicron variant infection (subgroup of age<65)

Odds Ratio Estimates

Effect	Point Estimate	95% Wald Confidence Interval	
Vaccination status, Vaccinated vs Unvaccinated	0.291	0.271	0.311
History of SARS-CoV-2 molecular test, No vs Yes	0.9	0.847	0.957
Preventive Care, No vs Yes	1.08	1.016	1.149
Number of outpatient and virtual visits, 0 vs >=11	1.595	1.404	1.813
Number of outpatient and virtual visits, 1-4 vs >=11	1.417	1.311	1.531
Number of outpatient and virtual visits, 5-10 vs >=11	1.267	1.181	1.36
Obese, No vs Yes	1.033	0.976	1.093
Obese, Unknown vs Yes	0.991	0.906	1.084
Charlson comorbidity score, 0 vs >=2	1.127	0.992	1.281
Charlson comorbidity score, 1 vs >=2	1.038	0.903	1.193
Frailty, Quartile 1 vs Quartile 4	1.037	0.956	1.124
Frailty, Quartile 2 vs Quartile 4	1.056	0.965	1.155
Frailty, Quartile 3 vs Quartile 4	1.051	0.973	1.135
Specimen type, Nasopharyngeal/oropharyngeal swab vs Saliva	1.701	1.59	1.819
Immunocompromised status, No vs Yes	1.063	0.899	1.258
History of COVID-19 diagnosis, No vs Yes	1.491	1.391	1.599

Supplementary Table 28. Full model for 3-dose VE against omicron variant infection (subgroup of age≥65)

Odds Ratio Estimates			
Effect	Point Estimate	95% Wald Confidence Interval	
Vaccination status, Vaccinated vs Unvaccinated	0.357	0.283	0.45
History of SARS-CoV-2 molecular test, No vs Yes	1.117	0.931	1.341
Preventive Care, No vs Yes	0.793	0.573	1.098
Number of outpatient and virtual visits, 0 vs >=11	0.765	0.327	1.787
Number of outpatient and virtual visits, 1-4 vs >=11	1.089	0.783	1.515
Number of outpatient and virtual visits, 5-10 vs >=11	0.849	0.689	1.045
Obese, No vs Yes	0.869	0.727	1.04
Obese, Unknown vs Yes	0.732	0.432	1.238
Charlson comorbidity score, 0 vs >=2	1.147	0.911	1.444
Charlson comorbidity score, 1 vs >=2	1.034	0.827	1.294
Frailty, Quartile 1 vs Quartile 4	1.061	0.794	1.417
Frailty, Quartile 2 vs Quartile 4	1.076	0.834	1.389
Frailty, Quartile 3 vs Quartile 4	1.04	0.819	1.319
Specimen type, Nasopharyngeal/oropharyngeal swab vs Saliva	1.316	1.055	1.641
Immunocompromised status, No vs Yes	1.232	0.868	1.75
History of COVID-19 diagnosis, No vs Yes	1.136	0.827	1.56

Supplementary Table 29. Full model for 3-dose VE against omicron variant infection (subgroup of female)

Odds Ratio Estimates			
Effect	Point Estimate	95% Wald Confidence Interval	
Vaccination status, Vaccinated vs Unvaccinated	0.3	0.276	0.326
History of SARS-CoV-2 molecular test, No vs Yes	0.871	0.805	0.944
Preventive Care, No vs Yes	1.006	0.928	1.09
Number of outpatient and virtual visits, 0 vs >=11	1.556	1.294	1.87
Number of outpatient and virtual visits, 1-4 vs >=11	1.5	1.363	1.651
Number of outpatient and virtual visits, 5-10 vs >=11	1.251	1.151	1.359
Obese, No vs Yes	1.043	0.973	1.119
Obese, Unknown vs Yes	0.996	0.874	1.135
Charlson comorbidity score, 0 vs >=2	1.119	0.967	1.295
Charlson comorbidity score, 1 vs >=2	1.036	0.887	1.21
Frailty, Quartile 1 vs Quartile 4	0.991	0.89	1.102
Frailty, Quartile 2 vs Quartile 4	1.012	0.908	1.128
Frailty, Quartile 3 vs Quartile 4	1.096	0.994	1.209
Specimen type, Nasopharyngeal/oropharyngeal swab vs Saliva	1.578	1.45	1.716
Immunocompromised status, No vs Yes	1.204	0.986	1.472
History of COVID-19 diagnosis, No vs Yes	1.462	1.338	1.597

Supplementary Table 30. Full model for 3-dose VE against omicron variant infection (subgroup of male)

Odds Ratio Estimates			
Effect	Point Estimate	95% Wald Confidence Interval	
Vaccination status, Vaccinated vs Unvaccinated	0.3	0.271	0.334
History of SARS-CoV-2 molecular test, No vs Yes	0.975	0.895	1.061
Preventive Care, No vs Yes	1.165	1.061	1.279
Number of outpatient and virtual visits, 0 vs >=11	1.43	1.191	1.716
Number of outpatient and virtual visits, 1-4 vs >=11	1.245	1.101	1.407
Number of outpatient and virtual visits, 5-10 vs >=11	1.156	1.032	1.295
Obese, No vs Yes	0.978	0.898	1.066
Obese, Unknown vs Yes	0.946	0.839	1.068
Charlson comorbidity score, 0 vs >=2	1.093	0.932	1.282
Charlson comorbidity score, 1 vs >=2	0.983	0.827	1.169
Frailty, Quartile 1 vs Quartile 4	1.113	0.986	1.256
Frailty, Quartile 2 vs Quartile 4	1.111	0.966	1.278
Frailty, Quartile 3 vs Quartile 4	1.017	0.906	1.142
Specimen type, Nasopharyngeal/oropharyngeal swab vs Saliva	1.78	1.612	1.966
Immunocompromised status, No vs Yes	0.978	0.776	1.233
History of COVID-19 diagnosis, No vs Yes	1.476	1.328	1.641

Supplementary Table 31. Full model for 3-dose VE against omicron variant infection (subgroup of Hispanic)

Odds Ratio Estimates			
Effect	Point Estimate	95% Wald Confidence Interval	
Vaccination status, Vaccinated vs Unvaccinated	0.32	0.29	0.354
History of SARS-CoV-2 molecular test, No vs Yes	0.844	0.772	0.922
Preventive Care, No vs Yes	1.078	0.989	1.176
Number of outpatient and virtual visits, 0 vs >=11	1.615	1.35	1.932
Number of outpatient and virtual visits, 1-4 vs >=11	1.405	1.26	1.567
Number of outpatient and virtual visits, 5-10 vs >=11	1.228	1.112	1.356
Obese, No vs Yes	1.009	0.933	1.09
Obese, Unknown vs Yes	1.045	0.919	1.188
Charlson comorbidity score, 0 vs >=2	1.112	0.943	1.31
Charlson comorbidity score, 1 vs >=2	1.081	0.908	1.287
Frailty, Quartile 1 vs Quartile 4	1.027	0.914	1.153
Frailty, Quartile 2 vs Quartile 4	1.031	0.909	1.169
Frailty, Quartile 3 vs Quartile 4	1.134	1.016	1.265
Specimen type, Nasopharyngeal/oropharyngeal swab vs Saliva	1.655	1.496	1.83
Immunocompromised status, No vs Yes	1.067	0.847	1.343
History of COVID-19 diagnosis, No vs Yes	1.549	1.415	1.696

Supplementary Table 32. Full model for 3-dose VE against omicron variant infection (subgroup of Non-Hispanic and others)

Odds Ratio Estimates			
Effect	Point Estimate	95% Wald Confidence Interval	
Vaccination status, Vaccinated vs Unvaccinated	0.286	0.262	0.312
History of SARS-CoV-2 molecular test, No vs Yes	0.978	0.905	1.056
Preventive Care, No vs Yes	1.067	0.981	1.161
Number of outpatient and virtual visits, 0 vs >=11	1.519	1.281	1.802
Number of outpatient and virtual visits, 1-4 vs >=11	1.35	1.218	1.496
Number of outpatient and virtual visits, 5-10 vs >=11	1.187	1.085	1.299
Obese, No vs Yes	1.025	0.95	1.106
Obese, Unknown vs Yes	0.933	0.826	1.053
Charlson comorbidity score, 0 vs >=2	1.075	0.933	1.239
Charlson comorbidity score, 1 vs >=2	0.939	0.805	1.096
Frailty, Quartile 1 vs Quartile 4	1.093	0.979	1.22
Frailty, Quartile 2 vs Quartile 4	1.092	0.97	1.231
Frailty, Quartile 3 vs Quartile 4	1.074	0.97	1.189
Specimen type, Nasopharyngeal/oropharyngeal swab vs Saliva	1.666	1.532	1.811
Immunocompromised status, No vs Yes	1.131	0.925	1.382
History of COVID-19 diagnosis, No vs Yes	1.379	1.245	1.528

Supplementary Table 33. Full model for 3-dose VE against omicron variant infection (subgroup of immunocompromised population)

Odds Ratio Estimates			
**Effect**	**Point Estimate**	**95% Wald Confidence Interval**	
Vaccination status, Vaccinated vs Unvaccinated	0.706	0.5	0.997
History of SARS-CoV-2 molecular test, No vs Yes	1.271	0.899	1.798
Preventive Care, No vs Yes	1.62	1.088	2.414
Number of outpatient and virtual visits, 0 vs >=11	0.538	0.097	2.983
Number of outpatient and virtual visits, 1-4 vs >=11	1.174	0.644	2.141
Number of outpatient and virtual visits, 5-10 vs >=11	1.407	0.967	2.046
Obese, No vs Yes	0.947	0.703	1.275
Obese, Unknown vs Yes	0.732	0.256	2.095
Charlson comorbidity score, 0 vs >=2	1.03	0.708	1.501
Charlson comorbidity score, 1 vs >=2	0.853	0.561	1.297
Frailty, Quartile 1 vs Quartile 4	1.15	0.711	1.859
Frailty, Quartile 2 vs Quartile 4	1.336	0.857	2.082
Frailty, Quartile 3 vs Quartile 4	1.316	0.868	1.996
Specimen type, Nasopharyngeal/oropharyngeal swab vs Saliva	1.161	0.796	1.693
History of COVID-19 diagnosis, No vs Yes	1.413	0.91	2.193
Age group, 18-44 years old vs 75 and older	0.595	0.309	1.145
Age group, 45-64 years old vs 75 and older	0.902	0.482	1.688
Age group, 65-74 years old vs 75 and older	1.27	0.629	2.563
Sex, Female vs Male	1.033	0.764	1.396
Race/ethnicity, Hispanic vs Other/Unknown	1.122	0.542	2.319
Race/ethnicity, Non-Hispanic Asian vs Other/Unknown	1.021	0.422	2.471
Race/ethnicity, Non-Hispanic Black vs Other/Unknown	1.083	0.488	2.402
Race/ethnicity, Non-Hispanic White vs Other/Unknown	1.019	0.488	2.13

Supplementary Table 34. Full model for 3-dose VE against omicron variant infection (subgroup of non-immunocompromised)

Odds Ratio Estimates			
Effect	Point Estimate	95% Wald Confidence Interval	
Vaccination status, Vaccinated vs Unvaccinated	0.295	0.276	0.314
History of SARS-CoV-2 molecular test, No vs Yes	0.908	0.857	0.963
Preventive Care, No vs Yes	1.068	1.005	1.134
Number of outpatient and virtual visits, 0 vs >=11	1.57	1.389	1.774
Number of outpatient and virtual visits, 1-4 vs >=11	1.378	1.28	1.484
Number of outpatient and virtual visits, 5-10 vs >=11	1.21	1.132	1.294
Obese, No vs Yes	1.015	0.962	1.071
Obese, Unknown vs Yes	0.978	0.897	1.066
Charlson comorbidity score, 0 vs >=2	1.116	0.999	1.247
Charlson comorbidity score, 1 vs >=2	1.03	0.914	1.16
Frailty, Quartile 1 vs Quartile 4	1.064	0.984	1.151
Frailty, Quartile 2 vs Quartile 4	1.063	0.975	1.16
Frailty, Quartile 3 vs Quartile 4	1.087	1.01	1.17
Specimen type, Nasopharyngeal/oropharyngeal swab vs Saliva	1.705	1.6	1.817
History of COVID-19 diagnosis, No vs Yes	1.478	1.382	1.581
Age group, 18-44 years old vs 75 and older	0.37	0.307	0.445
Age group, 45-64 years old vs 75 and older	0.526	0.439	0.632
Age group, 65-74 years old vs 75 and older	0.832	0.685	1.009
Sex, Female vs Male	1.141	1.084	1.2
Race/ethnicity, Hispanic vs Other/Unknown	0.968	0.882	1.063
Race/ethnicity, Non-Hispanic Asian vs Other/Unknown	1.472	1.297	1.67
Race/ethnicity, Non-Hispanic Black vs Other/Unknown	0.931	0.83	1.044
Race/ethnicity, Non-Hispanic White vs Other/Unknown	1.015	0.921	1.119

Supplementary Table 35. Full model for 3-dose VE against omicron variant infection among individuals without COVID-19 history, age<65

Odds Ratio Estimates			
Effect	Point Estimate	95% Wald Confidence Interval	
Vaccination status, Vaccinated vs Unvaccinated	0.299	0.279	0.32
History of SARS-CoV-2 molecular test, No vs Yes	0.898	0.844	0.955
Preventive Care, No vs Yes	1.087	1.016	1.163
Number of outpatient and virtual visits, 0 vs >=11	1.577	1.373	1.81
Number of outpatient and virtual visits, 1-4 vs >=11	1.439	1.323	1.566
Number of outpatient and virtual visits, 5-10 vs >=11	1.28	1.185	1.382
Obese, No vs Yes	1.026	0.965	1.092
Obese, Unknown vs Yes	0.974	0.885	1.072
Charlson comorbidity score, 0 vs >=2	1.151	1.003	1.322
Charlson comorbidity score, 1 vs >=2	1.049	0.901	1.22
Frailty, Quartile 1 vs Quartile 4	1.032	0.946	1.126
Frailty, Quartile 2 vs Quartile 4	1.062	0.962	1.172
Frailty, Quartile 3 vs Quartile 4	1.052	0.968	1.142
Specimen type, Nasopharyngeal/oropharyngeal swab vs Saliva	1.727	1.607	1.855
Immunocompromised status, No vs Yes	1.035	0.865	1.239
Age Group, 18-44 years old vs 45-64 years old	0.697	0.653	0.744
Sex, Female vs Male	1.144	1.079	1.212
Race/ethnicity, Hispanic vs Other/Unknown	0.98	0.885	1.085
Race/ethnicity, Non-Hispanic Asian vs Other/Unknown	1.475	1.285	1.694
Race/ethnicity, Non-Hispanic Black vs Other/Unknown	0.927	0.816	1.052
Race/ethnicity, Non-Hispanic White vs Other/Unknown	1.027	0.923	1.143

Supplementary Table 36. Full model for 3-dose VE against omicron variant infection among individuals without COVID-19 history, age≥65

Odds Ratio Estimates			
Effect	Point Estimate	95% Wald Confidence Interval	
Vaccination status, Vaccinated vs Unvaccinated	0.355	0.279	0.451
History of SARS-CoV-2 molecular test, No vs Yes	1.14	0.95	1.368
Preventive Care, No vs Yes	0.791	0.564	1.109
Number of outpatient and virtual visits, 0 vs >=11	0.774	0.326	1.842
Number of outpatient and virtual visits, 1-4 vs >=11	1.034	0.738	1.449
Number of outpatient and virtual visits, 5-10 vs >=11	0.864	0.699	1.069
Obese, No vs Yes	0.836	0.695	1.006
Obese, Unknown vs Yes	0.715	0.417	1.226
Charlson comorbidity score, 0 vs >=2	1.182	0.933	1.497
Charlson comorbidity score, 1 vs >=2	1.001	0.794	1.262
Frailty, Quartile 1 vs Quartile 4	1.009	0.748	1.362
Frailty, Quartile 2 vs Quartile 4	1.082	0.833	1.405
Frailty, Quartile 3 vs Quartile 4	1.029	0.806	1.316
Specimen type, Nasopharyngeal/oropharyngeal swab vs Saliva	1.33	1.061	1.669
Immunocompromised status, No vs Yes	1.149	0.802	1.646
Age group, 65-74 years old vs 75 and older	0.846	0.694	1.031
Sex, Female vs Male	0.945	0.795	1.124
Race/ethnicity, Hispanic vs Other/Unknown	0.996	0.588	1.688
Race/ethnicity, Non-Hispanic Asian vs Other/Unknown	1.037	0.59	1.823
Race/ethnicity, Non-Hispanic Black vs Other/Unknown	0.899	0.511	1.58
Race/ethnicity, Non-Hispanic White vs Other/Unknown	0.952	0.566	1.6
