# Supplementary material for: Effectiveness of mRNA-1273 against SARS-CoV-2 Omicron and Delta variants
Source: Nat Med. 2022 Feb 21;28(5):1063–71. doi: 10.1038/s41591-022-01753-y (PMC9117141; doi:10.1038/s41591-022-01753-y)
Supplement: Supplementary file 2 — Reporting Summary [file 41591_2022_1753_MOESM2_ESM.pdf]

nature portfolio

Corresponding author(s): Hung Fu Tseng

Last updated by author(s): February 16, 2022

Reporting Summary

Nature Portfolio wishes to improve the reproducibility of the work that we publish. This form provides structure for consistency and transparency in reporting. For further information on Nature Portfolio policies, see our Editorial Policies and the Editorial Policy Checklist.

Statistics

For all statistical analyses, confirm that the following items are present in the figure legend, table legend, main text, or Methods section.

n/a	Confirmed	
☐	☒	The exact sample size (*n*) for each experimental group/condition, given as a discrete number and unit of measurement
☒	☐	A statement on whether measurements were taken from distinct samples or whether the same sample was measured repeatedly
☐	☒	The statistical test(s) used AND whether they are one- or two-sided<br>*Only common tests should be described solely by name; describe more complex techniques in the Methods section.*
☐	☒	A description of all covariates tested
☐	☒	A description of any assumptions or corrections, such as tests of normality and adjustment for multiple comparisons
☐	☒	A full description of the statistical parameters including central tendency (e.g. means) or other basic estimates (e.g. regression coefficient) AND variation (e.g. standard deviation) or associated estimates of uncertainty (e.g. confidence intervals)
☒	☐	For null hypothesis testing, the test statistic (e.g. *F*, *t*, *r*) with confidence intervals, effect sizes, degrees of freedom and *P* value noted<br>*Give P values as exact values whenever suitable.*
☒	☐	For Bayesian analysis, information on the choice of priors and Markov chain Monte Carlo settings
☒	☐	For hierarchical and complex designs, identification of the appropriate level for tests and full reporting of outcomes
☒	☐	Estimates of effect sizes (e.g. Cohen's *d*, Pearson's *r*), indicating how they were calculated

Our web collection on statistics for biologists contains articles on many of the points above.

Software and code

Policy information about availability of computer code

Data collection	SAS v 9.4
Data analysis	SAS v 9.4 The code is available at GitHub: https://github.com/YiXLuo/P901-Omicron-Manuscript---Nature-Medicine

For manuscripts utilizing custom algorithms or software that are central to the research but not yet described in published literature, software must be made available to editors and reviewers. We strongly encourage code deposition in a community repository (e.g. GitHub). See the Nature Portfolio guidelines for submitting code & software for further information.

Data

Policy information about availability of data

All manuscripts must include a data availability statement. This statement should provide the following information, where applicable:

- Accession codes, unique identifiers, or web links for publicly available datasets
- A description of any restrictions on data availability
- For clinical datasets or third party data, please ensure that the statement adheres to our policy

Individual-level data reported in this study are not publicly shared. Upon request, and subject to review, KPSC may provide the deidentified aggregate-level data that support the findings of this study. Deidentified data (including participant data as applicable) may be shared upon approval of an analysis proposal and a signed data access agreement.

Field-specific reporting

Please select the one below that is the best fit for your research. If you are not sure, read the appropriate sections before making your selection.

☒ Life sciences ☐ Behavioural & social sciences ☐ Ecological, evolutionary & environmental sciences

For a reference copy of the document with all sections, see nature.com/documents/nr-reporting-summary-flat.pdf

Life sciences study design

All studies must disclose on these points even when the disclosure is negative.

Sample size	The study included 26,683 test-positive cases and 67,847 test-negative controls, for a total 94,530 individuals.
Data exclusions	Individuals were excluded if they received a COVID-19 vaccine other than mRNA-1273, any dose of mRNA-1273 <14 days before the specimen collection date, 2 or 3 doses of mRNA-1273 <24 days apart from previous dose or >3 doses of mRNA-1273 prior to the specimen collection date. Additional exclusions included a positive SARS-CoV-2 test or COVID-19 diagnosis code ≤90 days before the specimen collection date.
Replication	Results can be replicated with deidentified data (including participant data as applicable) upon approval of an analysis proposal and a signed data access agreement.
Randomization	This is an observational study with no intervention randomization.
Blinding	This is an observational study in which the exposure (vaccine) was given under routine clinical practices. There is no blinding in the study.

Reporting for specific materials, systems and methods

We require information from authors about some types of materials, experimental systems and methods used in many studies. Here, indicate whether each material, system or method listed is relevant to your study. If you are not sure if a list item applies to your research, read the appropriate section before selecting a response.

Materials & experimental systems

n/a	Involved in the study	
☒	☐	Antibodies
☒	☐	Eukaryotic cell lines
☒	☐	Palaeontology and archaeology
☒	☐	Animals and other organisms
☐	☒	Human research participants
☒	☐	Clinical data
☒	☐	Dual use research of concern

Methods

n/a	Involved in the study	
☒	☐	ChIP-seq
☒	☐	Flow cytometry
☒	☐	MRI-based neuroimaging

Human research participants

Policy information about studies involving human research participants

Population characteristics	The study included total 94530 individuals. 54396 were females, 40134 were males; 39520 were unvaccinated individuals, 1477 received 1 dose, 38739 received 2 doses, and 14794 received 3 doses of mRNA-1273.
Recruitment	Participants included those who had ≥12 months of KPSC membership before the specimen collection date. Participants data were extracted from the KPSC integrated health care system. Cases included individuals who tested positive by the RT-PCR TaqPath COVID-19 kit, and had specimens collected between 12/6/2021 and 12/31/2021. Controls included all individuals who tested negative with specimens collected between 12/6/2021 and 12/31/2021 and with the same age and membership requirement as cases. Potential self-selection biases include, 1) The study may not be generalizable to people who are not tested, including those with milder symptoms who may not pursue testing; 2) Differential care/test-seeking behaviors between vaccinated and unvaccinated individuals may introduce biases.
Ethics oversight	The study was approved by KPSC Institutional Review Board. All study staff with access to protected health information were trained in procedures to protect the confidentiality of participant data. A waiver of informed consent was obtained as this is an observational study of authorized and recommended Moderna COVID-19 vaccine administered in the course of routine clinical care. To facilitate the conduct of this study, a waiver was obtained for written HIPAA authorization for research involving use of the EHR.

Note that full information on the approval of the study protocol must also be provided in the manuscript.
